# Performance Expectancies Moderate the Effectiveness of More or Less Generative Activities Over Time

**DOI:** 10.3389/fpsyg.2019.01623

**Published:** 2019-08-21

**Authors:** Marc-André Reinhard, Sophia Christin Weissgerber, Kristin Wenzel

**Affiliations:** Department of Psychology, University of Kassel, Kassel, Germany

**Keywords:** desirable difficulties, problem-solving, generation effect, worked-examples, performance expectancies, meta-cognition, long-term learning

## Abstract

We examined if the benefits of generation for long-term learning depend on individual differences in performance expectancies (PEs) prior to learning. We predicted that a greater generative activity (problem-solving) compared to less generative activity (worked-examples) should be more effective for pupils with higher PEs, especially in the long run. As a comparison group for problem-solving, we implemented a special type of worked-examples that decreased engaging in self-explanations, because our main prediction focused on PEs moderating the long-term effectivity of less versus greater generative activities. We tested students’ immediate and delayed performance (after 3 months) using coherent curricular materials on linear functions in a sample of eighth graders (advanced school track). The results were partly in line with our predictions: Although we found no moderation of PE and generative activity, we obtained the predicted 3-way interaction of PE, generative activity, and time. Immediately, greater generative activity (problem-solving) was beneficial for pupils with higher PEs, while for pupils with lower PEs, problem-solving versus worked-examples did not differ. In the delayed test, this pattern reversed: for lower PEs, greater generative activity outperformed less generative activities, but there was no difference for higher PEs. Unexpectedly, the initial advantage of problem-solving for higher PEs could not be maintained, decreasing over three subsequent months, whereas the performance in the worked-example condition remained at a comparable level for higher PEs. The change in performance in the problem-solving condition for lower PEs was descriptively less pronounced than in the worked-example condition, but statistically not different. We further investigated the effects of problem-solving and worked-examples on changes in PEs after learning and after testing, hinting at gradual decrease in PEs and greater metacognitive accuracy in the problem-solving condition due to a reduction of overconfidence.

## Introduction

The idea to trouble a learner by a difficult learning task may appear strange. Intuitively, wouldn’t one ease the learning task to match the learner’s achievement prediction in hope of raising said learner’s achievement prospects? Yet, a growing body of research on a phenomenon dubbed “desirable difficulties” (e.g., Bjork, 1994; Bjork and Bjork, 2011) indeed supports such a seemingly odd learning approach. The label “desirable difficulties” subsumes various learning conditions which require considerable but manageable effort to foster long-term learning. Although the introduced difficulties may not be beneficial for the short term, overcoming the difficulties may induce desirable cognitive processes and strengthen memory, thus paying off in the long run (e.g., Bjork and Bjork, 1992, 2011; Bjork, 1994).

It is often theorized that such learning gains can be attributed to stimulations of cognitive processes that increase an understanding and deeper encoding of information, and that desirable difficulties anchor information in long-term memory (e.g., Bjork and Bjork, 1992; Bjork, 1994). The kind of processing required of a difficult learning task and the processing used by the learner are identified as two central aspects regarding the desirability of a difficulty (McDaniel and Butler, 2011): Interindividual learner’s characteristics and the learning task can moderate the beneficial effects of desirable difficulties on learning success. A small but growing body of research concerns this interplay; thus, one goal of the present study is to examine the role of interindividual differences in performance expectancies (PEs) prior to learning as a moderator for learning outcomes when studying with two different activities: either with problem-solving requiring greater generation activity to solve math problems, or with (a special type of) worked-examples requiring less generative activity since the solution and solution steps were explained. The explicit instructions on the solution steps decrease learners’ engagement in self-explanation and therefore lower learners’ generative activity, while still providing expert mental models. Our worked-examples function as comparison group to problem-solving. As such, our special worked-examples condition resembles more closely the common (re-) reading control group in research on generation (e.g., Bertsch et al., 2007) and testing effects (e.g., Kornell et al., 2012). Learning by (re-)reading can lead to overconfidence as unjustifiably high meta-cognitive judgments of one’s learning compared to actual learning outcomes (e.g., Karpicke and Blunt, 2011). In this sense, studying our worked-examples may convey the (mistaken) assumption that read information is already learned, even though learners may not be able to recall the information. Such an *illusion of competence* can be the consequence of undiagnostic cues whenever information is present during studying and absent but solicited at a performance test (Koriat, 1997; Koriat and Bjork, 2006).

Desirable difficulties can decrease learners’ illusion of competence (e.g., Karpicke et al., 2009; Diemand-Yauman et al., 2011) by decreasing the mismatch of cognitive processing during study and during testing (McDaniel and Butler, 2011). Test and retrieval experience in particular reduce competence illusions (Koriat and Bjork, 2006). Thus, experiencing difficulties during problem-solving as a test event requiring greater generative activity may challenge learners’ competence illusion and in turn increase metacognitive accuracy (especially beyond the accuracy of studying worked-examples, which learners did not have to solve or engage in much self-explanations). In particular, literature on self-regulation has emphasized the value of accurate metacognitions for the regulation of future learning behavior (e.g., Zimmerman, 2008). Thus, another goal of the present study concerns the effects of problem-solving as the incantation of generation on changes and accuracy in PEs prompted after learning and after testing as metacognitive assessments.

Our present paper follows two related lines of argumentation. First, we introduce the generation effect as a desirable difficulty and introduce how interindividual differences can play a moderating role for learning success. These considerations serve to build the case for PEs *prior* to learning as moderators for problem-solving requiring greater generation activity than our worked-examples. We then outline how PEs *after* learning and testing may function as metacognitive assessments. These later PEs are likely differentially affected by problem-solving in contrast to worked-examples regarding competence illusions, which would pose consequences for metacognitive accuracy. Thus, PEs should be more accurate after working on greater generative problem-solving tasks than after less generative tasks of simply studying already worked-examples (with explicit explanations on solution steps).

### The Generation Effect as Desirable Difficulty

The benefits of multiple desirable difficulties [e.g., generation effect, Bertsch et al. (2007); testing effect, Roediger and Karpicke (2006); distributed learning, Cepeda et al. (2006)] for memory, comprehension, and transfer are well documented (e.g., Bertsch et al., 2007; Rowland, 2014; Adesope et al., 2017). One form of desirable difficulties is the generation effect, which concerns the finding that actively generated information (e.g., solving problems, finding solutions to problems, generating answers, or producing of information) is remembered better than if the same information is more passively consumed (e.g., reading already solved problems or already worked-examples; e.g., Bertsch et al., 2007). All generative activities have in common that they require learners to engage in more effortful and deeper processing. In line with this, generated information requires learners to go beyond the information, for instance by relational processing of the provided information or by constructing links to previous knowledge (see Wittrock, 1989; Fiorella and Mayer, 2016). In line with this, actively generating information is more difficult than its mere reception (e.g., McDaniel et al., 1988; Ebbinghaus, 1913; DeWinstanley and Bjork, 2004; Bertsch et al., 2007), as is the generation of predictions and inferences rather than repetitions of solutions (e.g., Crouch et al., 2004).

Despite – or actually because of – being more difficult, self-generation can be more effective (e.g., Bertsch et al., 2007). Beneficial generation effects in learning were shown with naturalistic and/or curricular materials regarding complex topics (e.g., astronomy, engineering, physics) conducted in schools and universities (e.g., Renkl et al., 2002; Crouch et al., 2004; Richland et al., 2005; Moreno et al., 2009). Thus, positive effects of generation tasks arise in complex and realistic situations (and not only in laboratory settings using artificial or simple tasks). Furthermore, the generation effect is often thought to be related to the testing effect but considered to be broader in retrieval mode (e.g., Karpicke and Zaromb, 2010) requiring more elaborative in-depth processing (e.g., Bertsch et al., 2007; Rowland, 2014). Moreover, the advantage of generation/testing increases for longer time periods between the generation task and the criterion test of the learned information (e.g., Bertsch et al., 2007), even though generation, for example of problem-solutions, may be undesirable in the short-term at the beginning of knowledge acquisition when worked-examples are more desirable (Kalyuga et al., 2003). Even worked-examples can outperform testing activities long-term when previous knowledge is low and the materials are high in element-interactivity (van Gog and Kester, 2012; van Gog et al., 2015). However, our special worked-examples, serving as control group, violated an important guideline (Renkl, 2014): Reducing self-explanation diminishes the effectivity of worked-examples (e.g., Berthold and Renkl, 2009; Hefter et al., 2014). The goal was to increase the difference in generative activity across both learning conditions: Problem-solving required greater generation, whereas worked-examples prompted little generative activities due to providing expert problem-solving schemes with high instructional guidance. Thus, we did not expect a worked-example effect (e.g., Schworm and Renkl, 2006; see also Wittwer and Renkl, 2010). It was necessary to avoid comparing two learning conditions that both entailed highly generative elements to examine our proposed moderation of PE and long-term effectivity for generative activities. However, worked-examples reduce cognitive load and are advantageous during initial acquisition. Problem-solving is more effective later on – after learners’ expertise has increased (e.g., Renkl and Atkinson, 2003) – as well as for learners with greater previous knowledge (e.g., Kalyuga et al., 2001). This phenomenon is known as the *expertise-reversal effect* (e.g., Kalyuga et al., 2003; Kalyuga and Renkl, 2010; Spanjers et al., 2011).

Because of the difficulty of the generation task, learners can make errors while generating or fail to generate/solve problems at all (especially if they are forced to engage in such a challenging learning task; cf., Metcalfe and Kornell, 2007). The efficiency of generation, however, depends on the success of generation; more accurately, generated items lead to more learning success (e.g., Richland et al., 2005; Rowland, 2014). Thus, giving feedback and/or correcting errors moderate the benefits gained from generation tasks (e.g., Slamecka and Fevreiski, 1983; Pashler et al., 2005; Kang et al., 2007; Metcalfe and Kornell, 2007; Potts and Shanks, 2014; Metcalfe, 2017). Taking this into account, different learner characteristics potentially moderate the positive effects of generation tasks. This notion is echoed in other research (e.g., expertise reversal effect; Kalyuga et al., 2003; Kalyuga and Renkl, 2010; Spanjers et al., 2011). For instance, the expertise reversal effect states that some learning processes that prove beneficial for weaker learners or learners with lower previous knowledge (due to reduced working memory load) have no effect, or even detrimental effects, for stronger learners or learners with higher previous knowledge. Thus, it seems important to check for learner requirements or moderators that enhance the benefits of difficult learning conditions.

A hypothesis for when difficulties are desirable explicitly conceptualizes the moderating role of learners for difficulties to be desirable, specifically, the fit between learners’ characteristics and the generation task; the fit of the learning content and the type of generation task; and the fit of the generation task and the performance test are interrelated (e.g., McDaniel et al., 2002; McDaniel and Butler, 2011). Thus, they emphasize learner characteristics and prerequisites as moderators for the beneficial effects of desirable difficulties on learning success.

On the one hand, the authors (e.g., McDaniel et al., 2002; McDaniel and Butler, 2011) imply that desirable difficulties may be especially beneficial for learners with lower (cognitive) abilities. That is, difficulties could lead to cognitive processes and applications of effective strategies that learners would not have spontaneously used themselves. This in turn enhances learning, so desirable difficulties instigate compensatory processes. For instance, different studies implementing varying forms of desirable difficulties supported this assumption for the following abilities: lower general intelligence, lower structure building readers, and lower cognitive motivation (lower need for cognition; McDaniel et al., 2002; Brewer and Unsworth, 2012; Schindler et al., 2019).

On the other hand, researchers also implied that desirable difficulties can only increase learning if learners are able to fulfill the prerequisites of the difficult tasks. Hence, the effectivity of the desirable difficulties is tied to complementary preconditions between learners and tasks. For instance, studies showed higher previous knowledge and higher reading skills to be prerequisites for beneficial desirable difficulties (McNamara et al., 1996; McDaniel et al., 2002). McDaniel et al. (2002) supposed that less able readers had to use most of their processing capacities to correctly generate the items, so that they had no cognitive resources left to further process and encode the information.

These assumptions indicate that learner characteristics can moderate the beneficial effects of desirable difficulties in the above-mentioned two ways. However, the assessment of learner characteristics has so far not been exhaustive, meaning that further characteristics, for instance (cognitive-motivational) expectancies, are worthy to be explored.

### Performance Expectancies Prior to Learning as Moderator for the Generation Effect

One such learner characteristic worth examining may be performance expectancies (further PE/PEs). Expectancies are theorized to influence learners’ behavioral orientations as well as the intensity or persistence of learners’ behaviors and consequently their performance (e.g., Eccles, 1983; Eccles and Wigfield, 2002). PEs describe individuals’ subjective beliefs or ratings of how well one will perform in academic or achievement related tasks (e.g., Eccles, 1983; Eccles and Wigfield, 2002; Marshall and Brown, 2004) and could be related to or influenced by previous knowledge (for instance, higher previous knowledge could enhance the expectation to solve the same tasks). PEs are metacognitive predictions about future performances with motivational consequences: Such expectancies have been shown to be positively related to actual performance because they can shape the time and effort learners invest in tasks (e.g., Marshall and Brown, 2004; Schindler et al., 2016). PEs depend on an individual’s self-concept and the perceived difficulty of the learning task (e.g., Marshall and Brown, 2004; Dickhäuser and Reinhard, 2006). PEs only enhanced actual performance for difficult tasks but had no influence on performance on easy tasks [probably because these can be solved without further effort; e.g., Marshall and Brown (2004), Reinhard and Dickhäuser (2009)]. This should be especially relevant for desirable difficulties, which are inherently more difficult learning tasks.

Accordingly, generation tasks (and the required more intensive and deeper information processing) should be more effective for learners with higher PEs: Learners with higher PEs should better match the difficult generation tasks because they are more motivated to exert (cognitive) effort, time, and persistence. In contrast, low PEs can potentially reduce learners’ motivation and persistence while working on generation tasks because learners believe that they will not be able to solve the tasks. Further, higher PEs can be seen as a more relevant learner characteristic for (difficult) tasks in which participants must actually solve problems, in contrast to (easier) tasks in which they have to read worked-examples.

### Performance Expectancies After Learning and Testing as Metacognitive Assessments

The previous considerations focused on PEs – formed prior to working on a learning task – as a learner characteristic, which may function as a moderator for learning success. PE in this sense is identified as another potential moderator similar to other moderators discussed above, like previous knowledge. The difference of PE in comparison to these aforementioned moderators lies in the metacognitive nature of PE, whereas previous knowledge is cognitive in nature. Thus, a metacognitive performance judgment prior to learning may moderate learning success. This can be seen as one part of the story. The second part concerns how metacognitive judgments can act as a moderator for regulatory processes during and after learning, and therefore act as a mediator for learning success (e.g., Serra and Metcalfe, 2009). In this sense, PE – formed during or after learning and testing – may potentially be tied to metacognitive accuracy and metacognitive accuracy (in tandem with regulation accuracy) was shown to function as a mediator for learning success (e.g., Thiede et al., 2003). Therefore, we will briefly consider how solving problems opposed to studying problem solutions may influence metacognitive assessments and accuracy.

Metacognition – which refers to the knowledge of one’s own cognitive processes – can direct regulatory processes such as restudy choices (Dunlosky and Metcalfe, 2009). For example, problem-solving can improve the accuracy of judgments of learning (JOL) by decreasing performance overestimations (Baars et al., 2014, 2016). Accurately estimating and monitoring one’s performance are important educational outcomes because accurate metacognition effectively guides studying (Dunlosky and Lipko, 2007). Since metacognitive assessments guide learning, for example, by invested time (Son and Metcalfe, 2000), mental effort (Mihalca et al., 2017), or restudy decisions (Thiede et al., 2003; Dunlosky and Rawson, 2012), so do PEs influence time and effort allocations (Schindler et al., 2016). Since PEs describe individuals’ subjective performance beliefs (Marshall and Brown, 2004), they are (task-specific) metacognitive competence ratings and as such are a form of metacognitive judgment. PEs prompted after learning – similar to JOL prompted after learning – should be less influenced by the self-concept and instead should be more rooted in the experience of the actual learning task. Therefore, previously found effects of problem-solving versus worked-example studying on metacognitions and accuracy are likely to apply to PEs as well.

### Effects of Problem-Solving on Performance Expectancies as Metacognitive Assessments

In contrast to problem-solving, worked-examples can be seen as procedural solution scaffolds and are thereby mentally less taxing (in terms of working-memory load) and designed to ease schema construction (Sweller, 2006). However, such reduced difficulty (relative to problem-solving) can have metacognitive drawbacks in terms of conveying an illusion of competence after studying worked-examples (Baars et al., 2014, 2016), for example, when the content is currently accessible but will not be (completely) available later (e.g., Koriat and Bjork, 2005; Karpicke et al., 2009). Competence illusions during and after learning can negatively impact learning success: Overconfidence can lead to faulty regulation, such as early study termination or inaccurate selection of materials for restudy (Thiede et al., 2003; Dunlosky and Rawson, 2012). Overconfidence may also lead learners to underestimate the effort necessary to internalize correct and complete problem-solving schemas from worked-examples (Kant et al., 2017). Thus, experiencing difficulties while learning with problem-solving may challenges learner’s competence illusion, which may stimulate learners to engage in deeper and (cognitive) more effortful information processing (e.g., McNamara et al., 1996; Diemand-Yauman et al., 2011); and increase metacognitive accuracy in terms of predicted performance and actual performance (Baars et al., 2014); as well as, increase regulation accuracy in terms of selecting the right materials for restudy (Baars et al., 2016).

Multiple reasons are discussed as to why problem-solving can improve metacognitive accuracy. Baars et al. (2016) suggests that problem-solving as a generation activity allows learners to recall and test the quality of their acquired schema. They further capitalize on the idea of postdiction judgments (Griffin et al., 2009), which refers to the idea of utilizing test performance of a previously completed task as a cue on which to base judgments. Others suggest that encoding and retrieval fluency can influence metacognitive judgments (Agarwal et al., 2008; Pieger et al., 2017). All have the same implication that problem-solving entails more accurate cues on which to base metacognitive judgments, reducing overconfidence and increasing metacognitive accuracy (Kant et al., 2017).

The presented logic and previous findings of problem-solving versus studying worked-example on metacognitions and accuracy should also prove applicable to PEs: Learners may use the experienced difficulty of solving problems as opposed to reading worked-examples as a cue to lower their PEs, because the difficulty of solving problems may challenge learners’ competence illusion. In contrast, reading less difficult worked-examples may not challenge learners’ competence misconceptions. If so, learners in the problem-solving condition should decrease their PEs after the learning task and indicate more accurate PEs with respect to the later test outcome. Learners in the worked-example condition should not adjust their PEs. Hence, metacognitive accuracy should be improved in the problem-solving condition in contrast to worked-examples.

### The Present Study

The present work focuses on the generation effect and examines the potentially moderating role of learners’ initial PEs. Generation tasks are demanding tasks that require the recruitment of more cognitive capacities and deeper/more elaborate processing to solve the tasks and overcome the challenge. Thus, learners must exert more thinking, more time, and more effort to solve such tasks to reap their benefits. Hence, participants should be motivated and persistent, but this is not automatically the case for every learner. Regarding learner characteristics, PEs can lead to higher performance in achievement tasks through more allocation of resources like time, persistence, and effort. Thus, higher PEs can be seen as a fit between the generation tasks and learners’ abilities to cope with them. As mentioned above, a better fit between (cognitive) prerequisites of the task and (motivational-cognitive) characteristics of the learner is important for the effectiveness of such difficulties. Learners with higher PE are potentially more prone to exert and persist in more effortful processing.

Due to the above theoretical and empirical arguments, we propose the following hypotheses: (H1) We assume a two-way interaction between the condition (problem-solving vs. worked-example) and time (immediate vs. delayed). Performances in the worked-example condition should be higher in the immediate test, while the performances benefits of problem-solving should be apparent at the delayed test (time × condition). We also suppose a two-way interaction between the condition and PEs. (H2) Higher PEs should be more advantageous when solving problems compared to reading worked-examples (PE × condition). Since generation effects are desirable difficulties that often have greater delayed benefits rather than immediate benefits, we can assume a three-way interaction of condition × PE × time. (H3) The advantage of problem-solving for higher PEs should be more pronounced later in the delayed performance test rather than in the immediate test. Therefore, we predict a three-way interaction of PEs, condition, and time (PE × condition × time). We tested these hypotheses based on students’ immediate and delayed performance (after 3 months) using coherent curricular materials on linear functions in a sample of eighth graders (advanced school track) and measuring PEs prior to engaging in the learning task.

The present work also investigates the effects of problem-solving and worked-examples on PEs after learning as a competence-related form of metacognitive judgment. Since problem-solving can affect metacognitive assessments and accuracy by decreasing competence illusions, the difficulty of solving problems may challenge a learner’s initial performance overestimates. In contrast, a mere reading of problems and their solutions should align with a higher (misplaced) sense of competence (cognitive illusion), which should result in higher PEs for problem-information than for read-only.

We will thus test if the formation of more accurate PEs depends on active problem-solving required by the learning task: Initial PE prior to learning (and hence prior the experimental manipulation) should not differ, whereas during learning (and hence depending on the experimental learning condition), PEs in the solving condition should be lower compared to the worked-example condition. This difference should be eliminated once the problems of the performance test are completed by all (that is, also by worked-example learners), and pupils must indicate retrospectively how well they thought they did in the test (because all learners experienced the difficulty of problem-solving, in this case of the test problems).

Regarding later PEs prior to the second performance test 3 months later, it is possible that pupils base their PEs on their judgments of their performance after the first test. In this scenario, PEs prior to the second test may equal the post-test PEs. Another scenario may be that pupils remember the learning experience and base it on the experienced difficulty while learning, thus PEs prior to the second test may be lower in the problem-solving condition. In either case, we predicted an interaction effect of condition and time on metacognitive judgments of performance (H4). Moreover, calibration accuracy (a smaller difference between expected performance and actual performance) should be more precise for problem-solvers in contrast to worked-examples: If learners in the problem-solving condition decrease their PEs after the learning task, their PEs should be more accurate with respect to the later test outcome. Learners’ unadjusted PEs in the worked-example condition maintain a competence misconception and therefore should be less accurate (H5). We tested these hypotheses by additionally measuring PEs after learning, after the immediate performance test, and prior to the delayed performance test.

## Materials and Methods

### Participants and Design

Participants were children in the eighth grade of the secondary school track recruited from a school located in a medium-sized town in Germany. Written, full, informed consent was obtained from the principals, teachers, parents, and children,^1^ which resulted in an initial sample of *N* = 71. Not all participants were present at the first in-class session in school, nor at the second in-class session 3 months later, resulting in *n*_Session 1_ = 68 (41 females) and *n*_Session 2_ = 64 (39 females). This led to *n* = 61 pupils being present at both in-class sessions (32 in the worked-example condition and 29 in the problem-solving condition; mean age = 13.64 years, *SD* = 0.58). The participants were randomly assigned to either the experimental condition (problem-solving) or control condition (worked-examples). The randomization was successful as the condition was not related to gender distributions (Φ_T1_ = 0.10, *p* = 0.46) or to competence distributions indicated by previous math grade (Spearman’s ρ = −0.14, *p* = 0.30; *M*_worked–example_ = 3.38 (∼C), *SD* = 1.04; *M*_problem–solving_ = 3.03 (∼C), *SD* = 1.12; *F*(1,59) = 1.52, *p* = 0.22, $\eta_{\text{p}}^{2}$ = 0.03), or to previous knowledge (Point-biserial *r* = 0.05, *p* = 0.70; *M*_worked–example_ = 10.95, *SD* = 3.93; *M*_problem–solving_ = 11.32, *SD* = 3.48; *F*(1,59) = 0.15, *p* = 0.70, $\eta_{\text{p}}^{2}$ = 0.00).^2^ Thus, pupils in both conditions had similar prerequisites. The materials were pre-tested and adapted in a (different) sample of *n* = 30 eighth graders prior to administration of the materials in their final form in the current sample. The study was a 2(condition: solving vs. worked-examples) × 2(post-test time point: immediate vs. delayed) design with condition as between-subjects factor and post-test time-point as within-subjects factor. As a token of appreciation at the end of the study, the children received sweets and a small gift (puzzles) for their time and effort.

### Procedures

Prior to the study, the teachers had briefly introduced the topic of linear functions to the children. The children were novices and therefore they had very low previous knowledge. The teachers were instructed to omit any exercises that would be related to computing slopes and functions in their introductory teachings. Furthermore, the teachers handed short questionnaires to the children. They contained the measurements of multiple personality variables (not relevant to the proposed hypotheses in this paper but covered in another manuscript on the relationship of personality and long-term performance in a surprise test) and were collected by the researchers prior to the in-class session. All obtained data from the participants were pseudonymized based on number codes to allow subsequent matching of the data in both in-class sessions. Data collection in all school classes was conducted by the second author, supported by research assistants. All materials were paper–pencil contained in folders. All participants were allowed to use a calculator.

#### First In-Class Session

**FIGURE 1:**
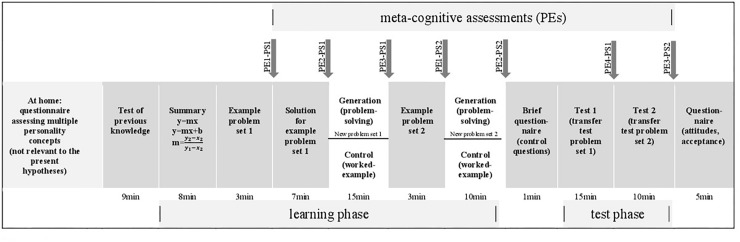
Timeline and schematic design in-class Session Time 1. Gray-colored arrays denote the same procedures and materials for all participants; white arrays show the differing procedures and materials according to the experimental manipulation. PE, performance expectancy; PT, problem set; EP, estimated performance; thus PE1-PS1, performance expectancy measurement 1 for problem set 1; PE2-PS1, performance expectancy measurement 2 for problem set 1; PE3-PS1, performance expectancy measurement 3 for problem set 1; PE1-PS2, performance expectancy measurement 1 for problem set 2; PE2-PS2, performance expectancy measurement 2 for problem set 2; EP1-PS1, estimated performance for problem set 1; EP1-PS2, estimated performance for problem set 2.

In class, participants were randomly re-seated. Multi-colored maps veiled the study’s condition to the children. The color-code also served to avoid having children with the same experimental condition clustered together. After a brief welcome to the pupils, all instructions were scripted, and all activities were timed. Figure 1 shows the procedures schematically. After a short test on participants’ previous knowledge (see Appendix Figure A1 in Supplementary Material), participants received two explanatory content pages (see Appendix Figure A2 in Supplementary Material). All participants were instructed to read the contents carefully, to try to comprehend them, and to keep in mind the important information highlighted in bold, bright red. They were told repeatedly that they would need the highlighted information in these explanatory materials for the upcoming test.

Once participants had studied the explanatory materials in their folders, they received a brief example of test problem set 1 (see Appendix Figure A3 in Supplementary Material). It had the same surface structure as in the upcoming test. Due to that, participants were asked to indicate their PEs for this problem set (PE1-PS1). Subsequently, all participants received the correct solution steps for this particularly presented example problem, followed by a second assessment of their PEs (PE2-PS1).

**FIGURE 2:**
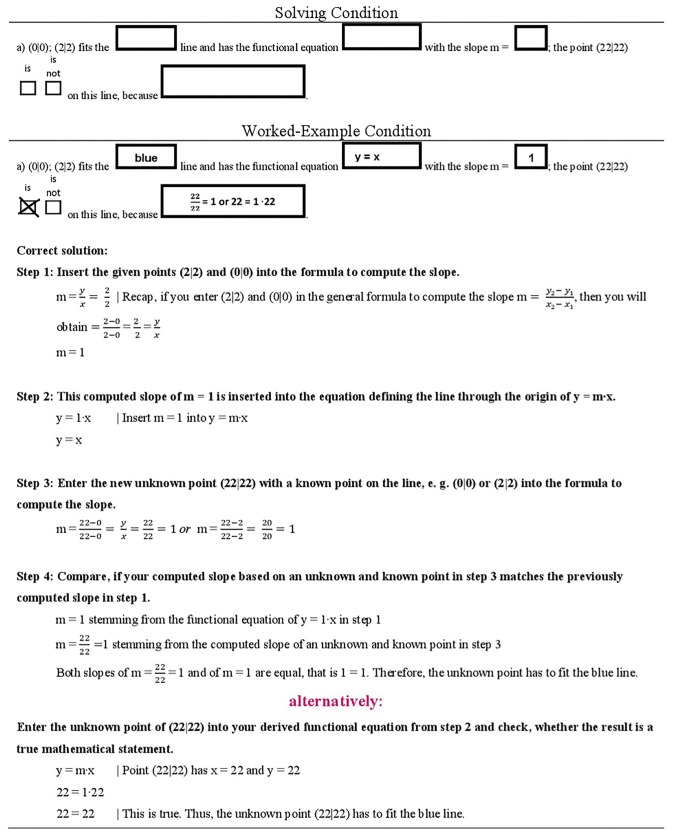
Learning phase: manipulation problem set 1.

The subsequent pages contained further problems of set 1, yet these problems differed for the participants depending on the experimental condition they were in (see Figure 2). In the worked-example condition, participants received these problems with all correct solution steps worked out, accompanied by short explanations of the steps; in the solving condition, participants had to solve all problems by themselves, however, they could refer to the previous materials (open-book). The instruction for participants in the worked-example condition read, “Please read the correct solution steps thoroughly, try to comprehend them, and learn them.” The instruction for the solving-condition read, “Please try to solve all problems.” Participants in the solving-condition were provided with the correct solutions for 2 min at the end of this task, then all participants were asked a third time to indicate their PEs for this problem set (PE3-PS1).

**FIGURE 3:**
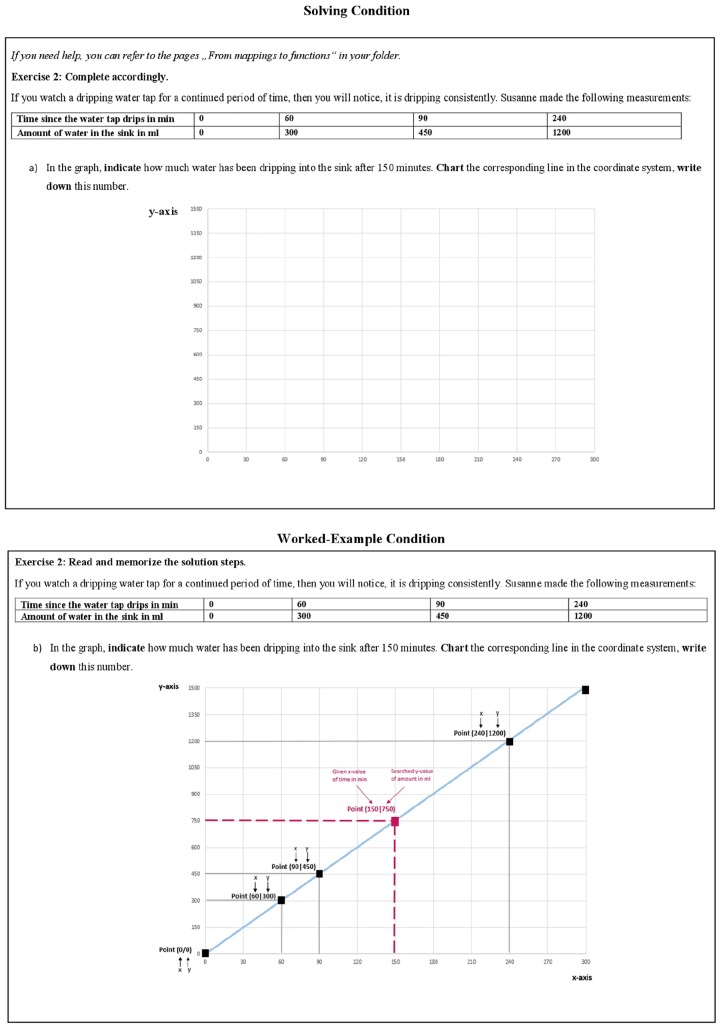
Learning phase: manipulation problem set 2.

The next page contained an example of a new problem set (problem set 2), which had a similar surface structure as in the upcoming test (see Appendix Figure A4 in Supplementary Material). Due to that, participants were asked to indicate their PEs (PE1-PS2). Again, the following pages differed for participants depending on their experimental condition (see Figure 3). In the worked-example condition, the correct solution steps were displayed with some explanations. In the solving conditions, the problem set had to be solved. Again, participants in the solving condition could use the previous materials as reference to help them solve the task, and they were provided with the correct solution for 2 min. For all participants, the next page contained the second PE measurement of problem set 2 (PE2-PS2). A short survey with control questions (like perceived task difficulty or invested effort) concluded the learning phase prior to the test phase (see Appendix B in Supplementary Material).

Participants started the test phase with seven new problems of set 1 and 15 min time to solve them (see Appendix Figure A5 in Supplementary Material). Thereafter, participants had 30 s to estimate how well they had just performed (PE4-PS1). Participants then continued with new problems of set 2 and 10 min of time and afterward were asked once again to estimate how well they had just performed (PE3-PS2). Once the test phase was finished, they answered questions regarding their overall learning and test experience and about their attitudes toward the learning method.

#### Second In-Class Session

**FIGURE 4:**
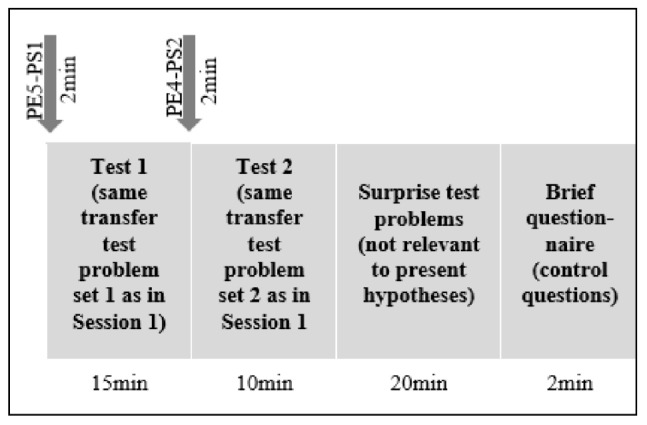
Timeline and schematic design in-class Session Time 2. PE4-PS1, performance expectancy measurement 4 for problem set 1; PE1-PS2, performance expectancy measurement 3 for problem set 2.

Both in-class sessions were 3 months apart (see Figure 4 for the schematic design of In-class Session 2). As in the previous session, participants were randomly re-seated. Once participants had opened their folders, they read that they would receive the exact same set of test problems as in Session 1 (pupils did not expect the second test). Yet, prior to the second test, they were again asked to indicate their PEs for the test problem set 1 (PE5-PS1) and for the test problem set 2 (PE3-PS2). Thereafter, pupils had 15 min time for the test problems of set 1 and 10 min time for the test problems of set 2. (Then, pupils had 20 min to solve the new surprise test problems, which were irrelevant to the hypothesis tested.) Session 2 concluded with a brief questionnaire with control questions (e.g., whether they took the test seriously and how much effort they invested in solving the problems). Finally, participants were thanked and dismissed.

### Materials and Measurements

Given the complexity of the materials, a few words on the materials’ structure and logic is warranted. The materials represented real curricular contents and were developed in cooperation with subject didactics. The contents focused on linear functions, specifically on computing slopes and deriving equations. Of the explanatory material (see Appendix Figure A2 in Supplementary Material), the first page pointed out similarities between a bijective mapping rule and the equation of a positive linear function. Both forms, *y* = *mx* (through the origin) and *y* = *mx* + *b* (shifted origin) were covered. The second page contained new content for the participants: the logic behind a slope and its formula for computation, the logic behind the *y*-axis and the constant b, and the link to the equation of a linear function. The materials of both problem sets in the learning phase focused on positive linear functions through the origin. Both test problems (see Appendix Figures A5, A6 in Supplementary Material) required transfer to negative linear functions. Both forms were required (*y* = *mx*; *y* = *mx* + *b*).

#### Problem Sets

Two coherent problem sets were chosen. Therefore, all following measurements and manipulations had to be phrased for both problem sets. For the analyses, like in any exam, one final score represented the test performance comprised of both problem sets.

##### Problem set 1

Problem set 1 required of the participants to (a) identify a line based on two given points in a coordinate system; (b) derive the functional equation; (c) compute the slope; (d) indicate whether a new point lies on the same line; and (e) proof the answer mathematically. Problem set 1 focused more on the execution of arithmetic computational procedures based on abstract contents.

##### Problem set 2

Problem set 2 required (a) sketching of a graph into a coordinate system; (b) finding a specific y-value in the graph; (c) explaining what a slope is; (d) computing the slope; (e) deriving the functional equation; and (f) computing a specific x-value. Problem set 2 focused more on the application of arithmetic formula to real-world contents.

#### Performance Expectancies

PEs were assessed as task-specific and therefore measured separately for each problem set (see Figure 1). After participants were shown an exemplary test problem of set 1 (see Appendix Figure A3 in Supplementary Material), three items recorded their PEs. The first item read, “*How well do you think you will perform in the upcoming test with this type of problems? Please estimate which grade you will be able to achieve in a test with seven test problems of this type.*” The range is from 1 = very good [A] to 6 = fail [F]). The second item read, “*How many points of 35 total do you think you will be able to achieve in the upcoming test?*” The range is from 0 to 35. The third item read, “*How many of the seven test problems of this type do you think you will be able to solve correctly in the upcoming test in 15-minutes of time?*” The range is from 1 to 7.^3^ PEs for problem set 2 were measured with two items (see Appendix Figure A4 in Supplementary Material).

In the test phase, after completing each test problem set, participants were asked to retrospectively estimate how well they *had* performed. The item for one’s post-test PEs (problem set 1) read, “*How well do you think you performed with respect to the previous test problems?*” Possible answers included, “*I think that I achieved _______ (grade).*”; “*I think I solved ____ (number) of seven problems correct*”; “*I think, I obtained ____ (points) of 35 points*.” The item for one’s post-test PEs of set 2 mirrored the items for problem set 1 (without the third item).

#### Experimental Manipulation

Figures 2, 3 illustrate the difference between both experimental conditions in the learning phase. In the Solving Condition, seven different problems of set 1 had to be solved. In the Worked-Example Condition, the same seven problems were presented along with their correct solutions, and along with each step necessary to solve the problem correctly (including short explanations). Likewise, in the Solving Condition, problem set 2 had to be solved by working alone, while in the worked-example condition the solutions and step-by-step guidance were provided. The instructions differed accordingly: “*Read, try to comprehend, and learn them*,” versus “*work out the solution by yourself*.” Participants in the solving conditions received the correct solutions to both problems for comparison.

#### Test Problems

The test problem sets had the same surface structure as the problems sets in the learning phase but required transfer (the problem sets for instance included only positive slopes and point of origins in (0| 0), whereas the slopes in the test problems were also negative and the points of origins could differ). Appendix Figures A5, A6 in Supplementary Material display all used test problems. The same test problem sets were used in Sessions 1 and 2. Two independent raters coded pupils’ answers to the test problems with high interrater-reliability (Session 1 *r* = 0.95 and Session 2 *r* = 0.97). Any remaining discrepancies were discussed and resolved. A total of 42 points could be achieved (with 35 points for problem set 1 and 7 points for problem set 2); Cronbach’s α = 0.88 (immediate post-test), Cronbach’s α = 0.92 (delayed post-test).

## Results

### Performance by Learning Conditions Across Post-tests

Exercising with worked-examples should be superior to problem-solving with respect to an immediate performance, but inferior to problem-solving in a later performance test (H1; see Table 1 for descriptive statistics). An rANOVA with time as within-subject factor and condition as between-subject factor (0 = worked-examples, 1 = problem-solving) tested this proposition. We found a main effect of time, *F*(1,59) = 9.34, *p* = 0.003, *MD* = −2.31, *SE* = 0.75, 95% CI [−3.81, −0.80], $\eta_{\text{p}}^{2}$ = 0.14, which means that the overall performance worsened by about 2 points. We found no main effect of condition, *F*(1,59) = 2.57, *p* = 0.11, *MD* = 2.43, *SE* = 1.52, $\eta_{\text{p}}^{2}$ = 0.04, 95% CI [−0.60, 5.47], only descriptively performances in the problem-solving condition (*M* = 15.75, *SE* = 1.10, 95% CI [13.55, 17.94]) was 2.43 points higher than in the worked-examples condition (*M* = 13.31, *SE* = 1.05, 95% CI [11.22, 15.41]). We obtained no interaction of time × condition, *F*[1, 59] = 0.83, *p* = 0.37, *B* = −1.37, *SE* = 1.51, 95% CI [−4.39, 1.6], $\eta_{\text{p}}^{2}$ = 0.01. Thus, there is no support for the proposed 2-way interaction of condition and time (H1).^4^

**TABLE 1:** Descriptive statistics of the central variables.

**Variable**	**Condition**			
	**Worked-examples**		**Problem-solving**	
	***M(SD)***	**95% CI**	***M(SD)***	**95% CI**
**In-class Session 1**				
Initial performance expectancy in points^4^	23.20 (7.94)	[20.27; 25.98]	18.87 (10.09)	[15.30; 22.35]
Test performance in points	14.13 (5.30)	[12.39; 16.08]	17.24 (6.85)	[15.07; 19.69]
**In-class Session 2**				
Test performance in points	12.50 (6.58)	[10.49; 14.90]	14.25 (7.62)	[11.81; 16.93]

*95% CI is based on bootstrapping with 1000 samples. All initial performance expectancies and both post-test points ranged from 0 to 42 points. ^4^When Initial performance expectancy is computed as mean of PS1-PE1 and PS1-PE2, summed with PS2-PE1 (that is with the initial performance expectancy after receiving an example solution averaged), the values are similar, *M* = 23.54, *SD* = 8.01, 95% CI [20.70, 26.19]. Performance expectancies prior and after seeing the example solution (PS1-PE1 and PS1-PE2) are not statistically different in both groups (Worked example: *t*(31) = −0.97, *p* = 0.34, *MD* = −0.67, *SD* = 3.91, 95% CI [−2.08, 0.74], *d*_*Cohen*_ = 0.09; Problem solving: *t*(28) = −1.45, *p* = 0.16, *MD* = −1.17, *SD* = 4.37, 95% CI [−2.83, 0.49], *d*_*Cohen*_ = 0.13).*

### Performance by Learning Conditions Across Post-tests Moderated by Performance Expectancies

The following analyses scrutinize whether the effectivity of both learning conditions differed as a function of post-test time point and (standardized) initial PEs (sum of PS1-PE1 and PS2-PE1). We examined whether learning with problem-solving was better for pupils with higher PEs (H2), especially in the long run (H3). All tests are reported two-tailed; the follow-up analyses as mean comparisons are conducted within the subsequent model and, if necessary, considered for higher (+1*SD*) and lower (−1*SD*) levels of standardized initial PEs and complemented by regions of significance (Johnson-Neyman technique; determined with PROCESS, Hayes, 2018).

We conducted repeated measures analyses of variance with time as within-subjects variable, condition as between-subjects variable (0 = worked-examples, 1 = problem-solving), and the standardized initial performance expectancy as a continuous moderator (cf. Judd et al., 2001) to specify the two-way and three-way interactions. We were predicting a two-way interaction of time × condition (H1), a two-way interaction of condition × initial performance expectancy (H2), as well as a three-way interaction of time × condition × initial performance expectancy (H3).

The results show a main effect of time, *F*(1,57) = 13.26, *p* < 0.001, *MD* = −2.70, *SE* = 0.74, 95% CI [−4.19, −1.22], $\eta_{\text{p}}^{2}$ = 0.19, a main effect of initial performance expectancy, *F*(1,57) = 19.83, *p* < 0.001, $\eta_{\text{p}}^{2}$ = 0.26, and a main effect of condition, *F*(1,57) = 8.17, *MD* = 3.89, *SE* = 1.36, *p* = 0.006, 95% CI [1.17, 6.52], $\eta_{\text{p}}^{2}$ = 0.13. Again, we did not obtain the expected interaction of time and condition (H1), *F*(1,57) = 0.24, *p* = 0.62, *MD* = −0.73, *SE* = 1.48, 95% CI [−3.70, 2.24], $\eta_{\text{p}}^{2}$ = 0.00. We found no convincing evidence for an interaction of initial performance expectancy and time, *F*(1,57) = 3.62, *p* = 0.06, $\eta_{\text{p}}^{2}$ = 0.06, and we did not find the predicted interaction of initial performance expectancy and condition (H2), *F*(1,57) = 0.08, *p* = 0.93, $\eta_{\text{p}}^{2}$ = 0.00; nevertheless, the postulated three-way interaction of time, initial performance expectancy, and condition was significant (H3), *F*(1,57) = 5.30, *p* = 0.025, *B* = −3.50, *SE* = 1.52, 95% CI [6.54, 0.46], $\eta_{\text{p}}^{2}$ = 0.09.^5^

**FIGURE 5:**
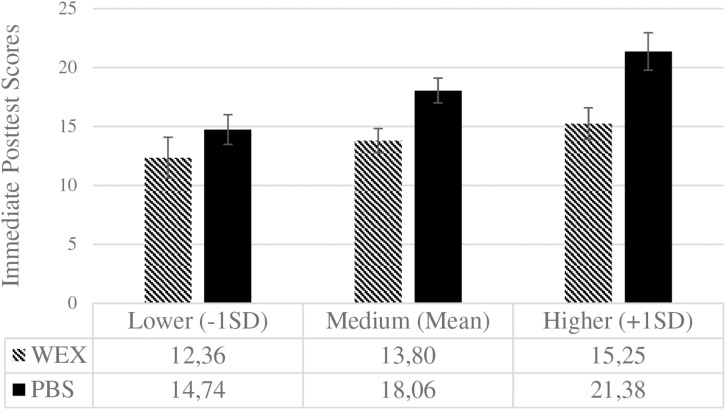
Immediate post-test scores for both learning conditions at different levels of initial performance expectancies. WEX, worked-examples (0), *n* = 32; PBS, problem-solving (1), *n* = 28. Error bars represent the standard error of the mean [WEX: 1.73 (–1SD), 1.02 (Mean), 1.33 (+1SD); PBS: 1.26 (–1SD), 1.06 (Mean), 1.58 (+1SD)]. Performance expectancies (standardized) are depicted for lower, medium, and higher levels. Post-test scores could range from 0 to 42.

**FIGURE 6:**
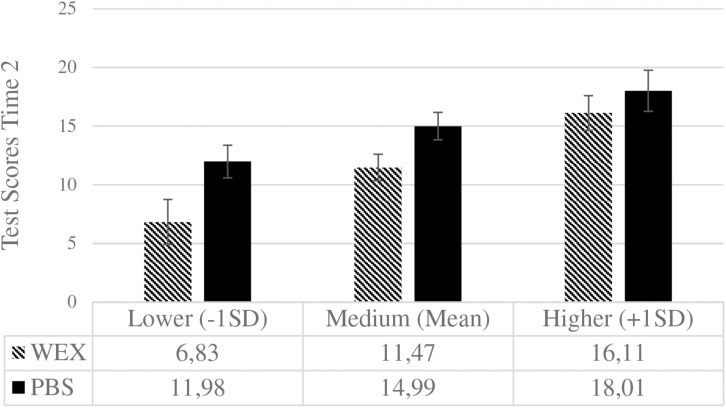
Delayed post-test scores for both learning conditions at different levels of initial performance expectancies. WEX, worked-examples (0), *n* = 32; PBS, problem-solving (1), *n* = 29. Error bars represent the standard error of the mean [WEX: 1.91 (–1SD), 1.13 (Mean), 1.48 (+1SD); PBS: 1.40 (–1SD), 1.17 (Mean), 1.75 (+1SD)]. Performance expectancies (standardized) are depicted for lower, medium, and higher levels. Post-test scores could range from 0 to 42.

**FIGURE 7:**
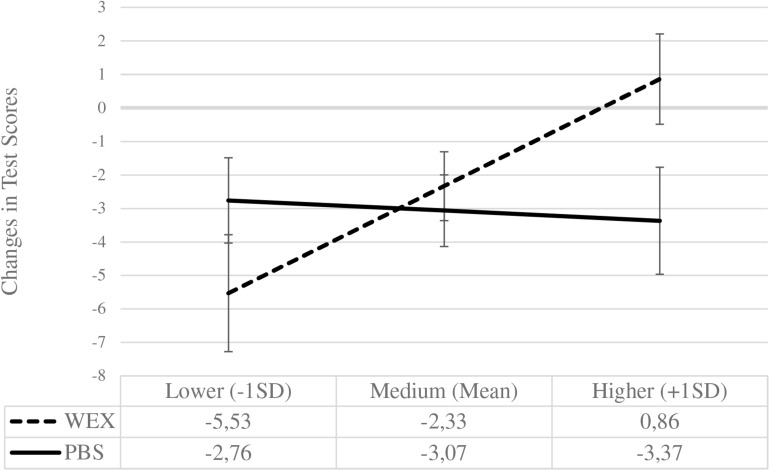
Performance changes across both post-test by learning condition and initial performance expectancies. Change scores on the *y*-axis were computed by subtracting the delayed post-test scores from the immediate post-test scores: Zero means no change, negative values mean performance loss, and positive values mean performance gains. The *x*-axis anchors these changes for both learning conditions (WEX, worked-example (0), *n* = 32; PBS, problem-solving (1), *n* = 29) for lower, medium, and higher levels of performance expectancies. Error bars represent the standard error of the mean [WEX: 1.74 (–1SD), 1.03 (Mean), 1.35 (+1SD); PBS: 1.27 (–1SD), 1.07 (Mean), 1.60 (+1SD)].

To understand these findings, we first attend to the adjusted main effects (for pupils with average initial PEs), which can be interpreted as performance decreases across time by about 2.5 points. The higher the initial PEs, the better pupils performed, and the overall performance in the problem-solving condition was about 4 points higher than in the worked-example condition. Note that the main effects of time and condition are the adjusted effects under consideration of initial PEs and thus represent the effects for an average level of initial PEs. The middle of Figures 5–7 illustrates these time and condition effects. More specifically (and given an average level of initial PE), in the immediate post-test, pupils in the problem-solving condition achieved 4.26 point more than those in the worked-example condition, *MD* = 4.26, *SE* = 1.47, *p* = 0.005, 95% CI [1.31, 7.19], Cohen’s *d* = 0.76, which amounted to a 3.52 point advantage in the delayed post-test, *MD* = 3.52, *SE* = 1.63, *p* = 0.034, 95% CI [0.27, 6.78], Cohen’s *d* = 0.56. The lack of support for the time × condition interaction is due to statistically similar performance decline over time in both learning conditions, *MD* = −0.73, *SE* = 1.48, 95% CI [−3.70, 2.23], Cohen’s *d* = −0.13. In the worked-example condition, post-test performance decreased about 2.5 points over time, *MD* = −2.33, *SE* = 1.03, 95% CI [−4.39, −0.28], Cohen’s *d* = −0.39, but about 3 points in the problem-solving condition *MD* = −3.07, *SE* = 1.07, 95% CI [−5.21, −0.92], Cohen’s *d* = −0.52. When decomposing the three-way interaction in terms of the two-way interaction of PEs × condition for the immediate post-test and the delayed post-test, neither of the two-way interactions was significant (Immediate Post-test: *B* = −1.87, *SE* = 1.51, *t*(57) = −1.25, *p* = 0.22, 95% CI [−4.89, 1.14] $\eta_{\text{p}}^{2}$ = 0.03; Delayed Post-test: *B* = 1.62, *SE* = 1.67, *t*(57) = 0.98, *p* = 0.33, 95% CI [−1.71, 4.96], $\eta_{\text{p}}^{2}$ = 0.02. This is no surprise, as there was no overall PEs × condition effect. However, when looking at the beta-values for the 2-way interaction, their opposite algebraic sign is noticeable, showing a cross-over. As such, the three-way interaction is a result of this cross-over effect pattern.

In the immediate post-test (see Figure 5), the learning conditions did not differ for lower levels (−1*SD*) of initial PEs, *MD* = 2.38, *SE* = 2.14, *p* = 0.27, 95% CI [−1.89, 6.66], Cohen’s *d* = 0.29 but did so for higher levels (+1*SD*), *MD* = 6.13, *SE* = 2.07, *p* = 0.004, 95% CI [1.98, 10.27], Cohen’s *d* = 0.77. As such, problem-solving was beneficial for pupils with higher initial PEs in the immediate post-test.

This pattern reverses for the delayed post-test (see Figure 6): For lower initial PEs, problem-solving outperformed worked-examples, *MD* = 5.15, *SE* = 2.37, *p* = 0.034, 95% CI [0.41, 9.89], Cohen’s *d* = 0.56, but there was no difference for higher levels, *MD* = 1.90, *SE* = 2.30, *p* = 0.41, 95% CI [−2.68, 6.48], Cohen’s *d* = 0.22.

Now we will look at the change in post-test performance over time (see Figure 7). Those with lower initial PEs in the worked-example condition showed a significant performance decline, *MD* = −5.53, *SE* = 1.74, *p* = 0.002, 95% CI [−9.19, −2.04], Cohen’s *d* = −0.55, as did those in the problem-solving condition, *MD* = −2.76, *SE* = 1.27, *p* = 0.034, 95% CI [−5.31, −0.21], Cohen’s *d* = −0.39. Although the performance decline in the problem-solving condition appears less pronounced, statistically both are comparable, *MD* = −2.77, *SE* = 2.16, *p* = 0.21, 95% CI [−1.55, 7.09], Cohens’ *d* = 0.33.

For higher levels of initial PEs, those in the worked-example condition showed a comparable performance, *MD* = −0.86, *SE* = 1.35, *p* = 0.53, 95% CI [−1.84, 3.56], Cohen’s *d* = 0.11, while the performance declined in the problem-solving condition, *MD* = −3.37, *SE* = 1.59, *p* = 0.039, 95% CI [−6.57, −0.17], Cohen’s *d* = −0.38. These slopes in performance change were statistically significant, *MD* = −4.23, *SE* = 2.01, *p* = 0.047, 95% CI [−8.41, −0.05], Cohens’ *d* = −0.53.

The Johnson-Neyman region of significance for the moderator (PROCESS, Hayes, 2018): PEs had a significant effect on changes in performance scores across both post-tests for all pupils with a (standardized) PE score of greater than 0.96.

These findings can be interpreted in the following way: For pupils with higher PEs, problem-solving in contrast to worked-examples was more beneficial resulting in an initial performance advantage. However, this early performance advantage could not be maintained in the delayed test (that is, 3 months later). The decline in performance represents the greater performance losses for higher PEs in the problem-solving condition in contrast to the worked-example condition, where performance across time was stable.

For those with lower PEs, immediate performance was not enhanced differently from either learning condition, but pupils who had learned with problem-solving showed higher delayed test scores than pupils who had learned with worked-examples. Descriptively, this is due to less pronounced performance declines over time for problem-solving in contrast to worked-examples, although the rates of performance decline are statistically not different.

### Later Performance Expectancies Over Time as Metacognitive Assessments

**FIGURE 8:**
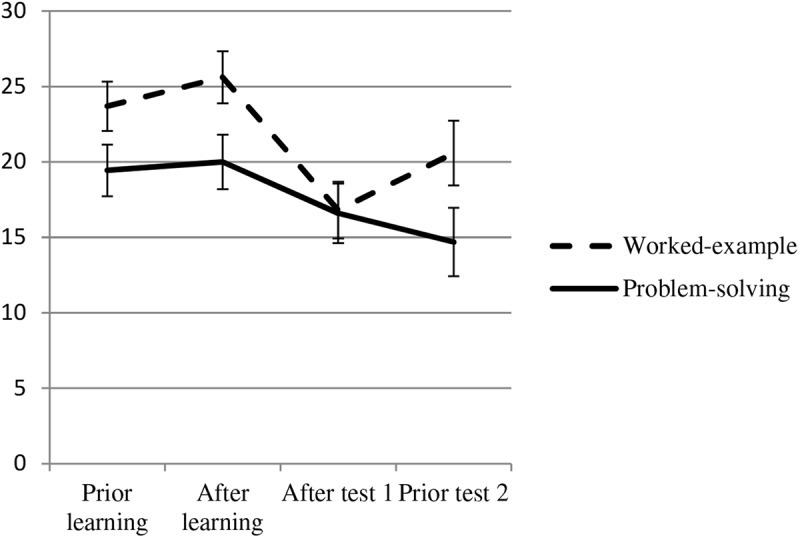
Change in performance expectancies by learning conditions. Error bars represent the standard error of the mean. Values could range from expecting 0 points to 42 points in the performance tests.

We argued that problem-solving may influence the resulting metacognitive PEs after learning and testing by reducing overconfidence, predicting an interaction of time × condition (H4). For the analyses (see Figure 8), we averaged the PEs (in points) measured after presenting the example test problem of type 1 and its solution (PE1-PS1, PE2-PS1). We summed up this value with the PEs of example test problem type 2 (PE1-PS2). The resulting value represents the PEs in points (from 0 to 42) *before* both problem types had been worked on differently due to the experimental conditions (that is, PEs *prior* to learning).^6^ We also summed up the PEs *after* learning with problem-type 1 and problem-type 2 (PE3-PS1, PE2-PS2; that is, PEs after learning). The same applies to the sum score of the post-test PEs for problem set 1 and problem set 2 *after* the first performance post-test (PE4-PS1, PE3-PS2 – that is, PEs after the immediate post-test at Session 1). At Session 2 and *prior* to the delayed performance test, the PEs for both problem types were summed up as well (PE5-PS1, PE4-PS2; that is, PEs prior the delayed post-test).

We subjected these indices of PEs to a repeated measures analysis of variance with condition as between-subject factor (2 levels: 0 = worked-example, 1 = problem-solving) and PEs (4 levels: prior to learning, after learning, after Test 1, prior Test 2) as within-subject variable (see Figure 8). Since the sphericity assumption was not met, we report the Greenhouse-Geisser-corrected *p*-values and degrees of freedom. This yielded a significant effect of time, *F*(2.28, 129.91) = 13.41, *p* < 0.001, $\eta_{\text{p}}^{2}$ = 0.19, but neither offered convincing evidence for a condition effect, *F*(1,57) = 3.02, *p* = 0.088, $\eta_{\text{p}}^{2}$ = 0.05, nor for the predicted interaction effect (H4), *F*(2.28, 129.91) = 2.61, *p* = 0.07, $\eta_{\text{p}}^{2}$ = 0.04.

We found little convincing support for H4. A reduction of PEs (and learner’s competence illusion) was only apparent as gradual change across assessment times (see Figure 8); with pre-existing differences (albeit non-significant) in the worked-example condition compared to the problem-solving condition. Thus, these results should be taken with caution.

### Calibration (Metacognitive Accuracy)

To obtain calibration (difference of predicted and actual test scores), we used the PEs (previously discussed in Figure 8) and the actual test scores: We computed a difference score of PEs prior to learning and immediate post-test performance; a difference score of PEs after learning and immediate post-test performance; a difference score of PEs after the immediate post-test and actual test performance in the immediate post-test; and, a difference score of later PEs prior the delayed post-test and actual performance in the delayed post-test. (Note, positive values denote overconfidence and negative ones underconfidence; Bugg and McDaniel, 2012).

Using these calibration values as dependent variables (within-subjects; 4 levels: calibration prior to learning, calibration after learning, calibration after the immediate post-test, calibration prior to the delayed post-test) and condition as independent variable (between-subjects) in an rANOVA yielded a main effect of condition, *F*(1,57) = 12.32, *MD* = −6.78, *SE* = 1.93, *p* < 0.001, 95% CI [−10.65, −2.91], $\eta_{\text{p}}^{2}$ = 0.18. Pupils in the worked-example group showed less accurate calibration and more overconfidence, *M* = 7.79, *SE* = 1.33, 95% CI [5.12, 10.45], while pupils’ calibrations in the problem-solving group was more accurate, *M* = 1.01, *SE* = 1.40, 95% CI [−1.80, 3.81]. Note that the calibration score of the problem-solving group is closer to 0, which denotes more accurate calibration, whereas a score of 7.79 in the worked-example group represents a difference of about 8 points between expectation and actual test scores.

**FIGURE 9:**
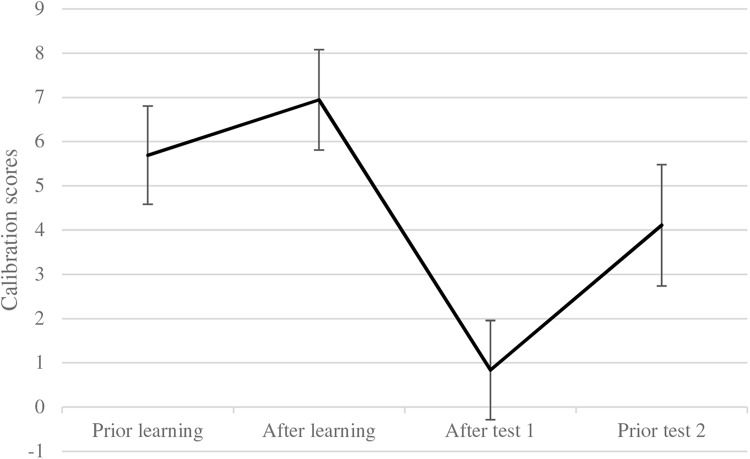
Main effect of calibration accuracy. Error bars represent the standard error of the mean.

We further found a main effect of calibration (reported with Greenhouse-Geisser correction), *F*(2.32,132.45) = 10.84, *p* < 0.001, $\eta_{\text{p}}^{2}$ = 0.16 (see Figure 9). Simple comparisons (Bonferroni-corrected) showed a significant difference of calibration *prior* to learning and calibration *after* the immediate post-test (*M* = 0.83, *SE* = 1.12), *MD* = 4.86, *SE* = 1.04, *p* < 0.001, 95% CI [2.01, 7.71] and a significant difference of calibration *after* learning and calibration *after* the immediate post-test, *MD* = 6.11, *SE* = 1.06, *p* < 0.001, 95% CI [3.20, 9.01]. This means calibration *after* the immediate post-test was more accurate than *prior* to and after learning. All other comparisons were not significant, all *p*s > 0.15. Finally, we did not find the expected interaction effect of calibration × condition, *F*(2.32,132.43) = 2.17, *p* = 0.11, 95% CI [2.99, 10.73], $\eta_{\text{p}}^{2}$ = 0.04. Overall this pattern indicates that the calibration in the problem-solving condition was more accurate than in the worked-example condition in general (but not as a consequence of the learning conditions or tests over time), and that calibration after the immediate test was more accurate than PEs prior to both tests. This pattern of results partially supports (H5). Overall calibration in the problem-solving condition was more accurate as in the worked-example condition showing overconfidence. However, due to the pre-existing differences (albeit non-significant) in initial PEs in the worked-example condition compared to the problem-solving condition (see Figure 8), the interpretation of the results on calibration due to overconfidence reduction is not routed in strong empirical evidence and should be taken with caution.

## Discussion

Our work examined learners’ PEs prior to learning as moderators for the effectiveness of different learning tasks (a special type of problem-solving vs. worked-examples) on immediate and delayed performance. The experiment was conducted in school and used curricular mathematical materials for learning. We assumed that the problem-solving condition would be superior to the worked-examples condition in the delayed post-test (time × condition; H1) and that problem-solving opposed to worked-examples are more beneficial for higher PEs (condition × PEs; H2). We further supposed that the moderating effect of PEs in the problem-solving condition would arise particularly in the delayed test (time × condition × PEs; H3). Moreover, we predicted an interaction effect of condition and time on metacognitive judgments of PEs measured after learning and testing (H4). Participants in the problem-solving condition (in comparison to participants in the worked-examples condition) should lower their PEs regarding the later test outcome after experiencing the difficult learning task (reduction of competence illusion). Finally, we also assumed that calibration accuracy (the difference between expected performance and actual performance) should be more precise for problem-solvers in contrast to participants in the worked-examples condition (H5). Participants in the worked-example condition probably maintain a competence misconception and thereby may have stronger differences between their expected and their actual performance. Thus, we expected initial PEs to be a moderator for learning performance and condition to be a moderator for later PEs, thus affecting metacognitive accuracy.

Our findings showed only a descriptive advantage of the problem-solving condition on the delayed learning performance (H1) and no two-way interaction of PEs and the condition (H2). However, taking into account prior PEs, we obtained a beneficial adjusted main effect of the problem-solving condition for participants with average PEs. Thus, problem-solving can be advantageous for certain learners. This is in line with the assumptions that PEs are only related to difficult (and not easy) tasks (like problem-solving) because difficult tasks require more effort, time, motivation, and persistence (e.g., Marshall and Brown, 2004; Reinhard and Dickhäuser, 2009). The obtained moderation supports the notion that learner characteristics are important for the effectiveness of desirable difficulties (e.g., McDaniel and Butler, 2011). For pupils with lower and average PEs, the problem-solving condition was more advantageous later on, while for higher PEs both learning conditions were equal at a delay. This is partly in line with the assumptions that the beneficial effects of generation tasks arise in the long run (e.g., Bjork and Bjork, 1992, 2011; Bjork, 1994): There was no significant interaction between time and condition, and only the consideration of initial PEs unveiled favorable effects at a delay. Without taking into account PEs, performance in the problem-solving condition was only descriptively better long-term; this could be due to the long delay between learning and the delayed test (this will be further discussed below).

The three-way interaction (PEs × time × condition) showed that participants with higher PEs in the problem-solving condition performed better in the immediate test, whereas participants with lower PEs in the problem-solving condition performed better in the delayed test. Unfortunately, higher PEs could not maintain this initial performance advantage in the problem-solving condition over time. Although participants with higher expectancies immediately profited from generation tasks, those with lower PEs also benefited from difficult tasks in the long run. Thus, as inquired in the beginning, it is not strange to trouble a learner who has lower PEs with hindered learning tasks. This is in line with the assumptions that desirable difficulties may be advantageous for learners with lower abilities or cognitive motivation (e.g., McDaniel et al., 2002; Schindler et al., 2019). It is important to note that these difficulties do not boost weaker learners’ performances to the level of stronger learners, but these difficulties prevent greater performance losses for weaker learners over time.

Overall, learners benefited in different manners from desirable difficulties. This fits previous work that was able to identify moderators (e.g., feedback, mood, previous knowledge, reading skills; e.g., McNamara et al., 1996; McDaniel et al., 2002; Bertsch et al., 2007; Schindler et al., 2017). The present findings also emphasize the importance of moderators for the effectiveness of generation activities.

When considering the effects of a generation activity on metacognitions, the results have to be taken with caution. A mere trend shows a gradual decrease in PEs in the problem-solving condition in contrast to the worked-examples condition over time (in which overconfident PEs did not change; H4); and a trend shows a pre-existing difference in PEs. The results show no convincing support for a learning event and time-driven overconfidence reduction (H4). Regarding our fifth hypotheses, our results showed a main effect of condition with greater metacognitive accuracy in the problem-solving condition than in the worked-examples condition (H5). Thus, calibration accuracy (the difference between expected performance and actual performance) was more precise for participants in the problem-solving condition in contrast to participants in the worked-examples condition. Yet, this interpretation is not routed in strong empirical evidence and should be taken with caution. These findings only hint that the problem-solving task may have led to a more realistic understanding of learners’ current competences and thus reduced participants’ competence illusion (e.g., Karpicke et al., 2009; Diemand-Yauman et al., 2011; Baars et al., 2014). Given the important role of accurate metacognitions for the regulation of learning (e.g., Dunlosky and Lipko, 2007), these findings nevertheless hint at the value of problem-solving.

The current study is not without some limitations, which will be discussed in the following section and which could be optimized in future work. We designed our study with real-world materials that were integrated within curricular content and natural math lesson progression. Although we coordinated with the teachers on what content was covered prior to our experimental session (introduction of the topic but no calculations), we had no control over actual implementations (although there were no differences in previous knowledge across both conditions). Moreover, after the first experimental in-class session, we had no control over any further progression of the lessons’ content, over homework or over subsequent topics, prior to the delayed test of Session 2. The teachers knew about the delayed test and were instructed not to repeat any content; however, we do not know what additional content with potential overlap was taught in the interim between Session 1 and Session 2, and/or what pupils learned in the meantime. Thus, although classroom studies are very important regarding work focusing on learning success, there are also many confounding factors that are not controllable, which presents a limitation. Performance in general was rather low, thus it would be interesting to extend the instructional units.

Another limitation relates to the fact that the tests in Sessions 1 and 2 were identical, thus the testing-effect may have played a role regarding learners’ performance, although likely not much given the 3-month delay. To avoid this, future studies may include one group tested immediately and another group only tested at a delay. In addition, our worked-examples included detailed explanations, so it may be that learners did not have to indulge in self-explanations (which can trigger the positive effects of worked-examples; e.g., Renkl et al., 1998). Hence, future research could use materials that require self-explanations. In line with this, it could also be that our problem-solving condition was superior to the worked-example condition not because of the generation task but because participants were presented with a shortened worked-example before the generation activity (see e.g., Paas, 1992), as well as briefly with the correct answers after the generation, and feedback is important for the effectivity (e.g., Slamecka and Fevreiski, 1983; Pashler et al., 2005; Kang et al., 2007; Metcalfe and Kornell, 2007; Potts and Shanks, 2014; Metcalfe, 2017). Thus, future studies could use different incantations of problem-solving tasks or worked-examples, all in the attempt to generalize findings and to try to optimize possible limitations due to our applied learning tasks. In line with this, in the applied problem-solving tasks students were able to look back at the explanations and introduction of the material given in the beginning (open book solving task). Although this, as well as later given feedback sheet regarding correct answers for the generation tasks, may have been beneficial, it is unclear to what extent students even used these aids. Some students may have never looked at the previous learning materials, whereas others may have relied on them often; some may have contemplated the correct solution steps after finding out a discrepancy in their results and the result provided on the answer sheet, others may have not. Although this is a typical occurrence in schools, future work could also try to manipulate how many times learners are able to look back at previously studied materials. Previous work also often implemented problem-solving tasks after worked-examples, thus combining these two strategies. In contrast, we compared sole problem-solving tasks and sole studying of worked-examples (both following a short introduction of the materials), so our methods are not completely in line with some of the above-mentioned literature. Future studies could thus explore the relation of PEs, problem solving following worked-examples, and long-time learning success.

A further, and possibly confounding or negative, aspect concerns the lag between post-test one and post-test two, which we set at 3 months. The 3-month lag taps into long-term learning but may have been too long given the overall low performance. Future research may include a shorter lag of only a few weeks. However, the choice of 3 months was implemented because we wanted to make sure that all teachers had finished the section on linear functions; naturally, the length of time dedicated to a topic depends on the teachers and on the class (in other words, some classes progress more quickly than others), which we cannot influence due to the field character of our study. In our case, we aimed for a comparable lag and for all teachers to have started new content so that the end-of-topic exam on linear functions did not coincidentally occur in temporal proximity to our delayed test. It would be valuable for future research to coordinate with teachers’ planned exam at the end of the session to include mutually agreed-upon exam questions that would also serve as a delayed test. One related problem/aspect of that strategy (and our research) would be that any previous one-time intervention may be too weak to detect differences in delayed exam performance as it may be overshadowed by teachers’ and students’ own exam preparations (which we cannot control). Relatedly, a single-intervention study may have to be paired with a shorter lag, or multiple controlled interventions are required for longer lags. The difficulty here lies in the willingness of the teachers and parents to participate, given real-world constraints and concerns that these interventions could disrupt the classes and take away valuable teaching time. Future research may also conceptualize a paradigm in which trained teachers take over teaching for one to 3 weeks, with multiple, ongoing experimental interventions that conclude with a graded interim exam as a delayed test. This may present the challenge of finding willing institutions, teachers, and/or parents.

To thoroughly test moderators, larger samples are needed (which is often difficult to obtain in school contexts). Of course, our findings can be interpreted only for German students within the same age-range, the same educational school track, and for the same learning materials (and very strictly seen, only for this school). Due to that, future work using bigger and more diverse samples (as well as different materials) is important. The same applies to learners with different levels of previous knowledge: Future studies could use more known topics, assess previous knowledge, and include this factor in the analyses. To gain access to more participants, another option for future research may include extracurricular learning environments (e.g., instead of homework), which could be implemented either online or onsite. For instance, a study could deploy carefully designed learning modules on selected (additional) curricular content that is not part of current class curriculum within a given school year; this might allow the implementation of thorough experimental designs while proving attractive to learners and teachers as a supplemental training learning environment. All in all, as pointed out by Dunlosky et al. (2013), future research may attend in general more to an investigation of moderators of various desirable difficulties (e.g., previous knowledge, different skill levels) because their roles are still less known.

We should note that previous work often focused on the effectiveness of generation tasks regarding recall and/or memory of learned information through later tests assessing the same or similar information, but our tests mostly assessed transfer (instead of identical information). Thus, the underlying effects of the learning conditions could be different (e.g., Glogger-Frey et al., 2015). Prior research regarding transfer and intentionally aggravated learning tasks resulted in varying findings: Some studies found beneficial effects of desirable difficulties solely for identical or easy information but not for transfer (e.g., Lehmann et al., 2016) or that worked examples were more important for transfer (e.g., Glogger-Frey et al., 2015). In contrast, some studies found beneficial effects of desirable difficulties also for changed materials and transfer (see e.g., Dunlosky et al., 2013 for a good overview). Thus, future studies could implement transfer as well as identical questions.

As mentioned above, generation tasks reduce learners’ competence illusion and overconfidence, thus participants in the problem-solving condition should be able to more accurately calibrate their PEs than do participants in the worked-example condition, who could still possess overconfident expectancies. Our findings only hint at this relationship. Participants’ PEs appeared to differ between the conditions before the learning tasks even started. This does not have to be an indicator that the randomization of our sample failed but could rather indicate that participants (unbeknown be us) checked the tasks and their condition by looking at the materials prior to the learning task, which serves as a limitation. Hence, their initial PEs could have been influenced by participants’ knowledge of the upcoming learning tasks.

## Conclusion

Our results emphasize the importance of moderators for the desirability of generation activities, and the desirability of generation activities for metacognitive outcomes. Regarding implications for the educational context, we still cannot recommend that teachers use or not use problem-solving tasks. Our work, though, is a step in the right direction, while more research exploring the effectiveness of problem-solving tasks or moderators are still needed. Thus, we underscore the value of longitudinal studies or studies using multiple learning phases as well as multiple learning success assessments for evidence-driven educational recommendations.

## Ethics Statement

This study was conducted in full accordance with the Ethical Guidelines of the German Association of Psychologists (DGPs), the American Psychological Association (APA), and the Hessian Ministry for Science and the Arts. The study was approved by the Hessian Ministry for Science and the Arts. Full consent was obtained of the principal, the teachers, parents and pupils.

## Author Contributions

M-AR provided funding, developed the basic idea on performance expectancies as moderator, and provided the critical comments. SW developed the basic idea of performance expectancies as metacognitions, developed the materials, contributed to the data collection and data preparation. M-AR and SW contributed to the study design and analyzed the data. SW and KW wrote and revised the manuscript.

## Conflict of Interest Statement

The authors declare that the research was conducted in the absence of any commercial or financial relationships that could be construed as a potential conflict of interest.

## Footnotes

1 This study was conducted in full accordance with the Ethical Guidelines of the German Association of Psychologists (DGPs) and the American Psychological Association (APA). This study was fully approved by the Ethics Committee affiliated with the Hessian Ministry for the Science and the Arts (Yasar Karakas, Hessisches Kultusministerium, Referat I.3.2, Luisenplatz 10, 65185 Wiesbaden, Germany; Phone: +49 611 368 – 2734; E-mail: Yasar.Karakas@kultus.hessen.de).

2 The pupils were novices to linear functions. Most points in the previous knowledge test were achieved based on recognizing the graphs of linear functions (Appendix Figure A1 in Supplementary Material, task 2); not by the tasks 4–7.

3 Note that we included three questions to assess performance expectancies because we were not sure about accuracy and variance of pupils’ judgments – whether eighth graders would naturally judge their performance in expected grades, or points, or number of test problems solved – and whether grade and number of test problems solved would vary enough for meaningful analyses. Since the three assessments were highly correlated and variances were highest for judgments in points, we included the performance expectancies of points in the main analyses. (Expected points also had the same metric as points achieved in both post-tests.)

4 The results are the same when including (standardized) previous knowledge or math grade in the model.

5 The results are the same when including (standardized) previous knowledge or math grade in the model. Thus, both previous knowledge and previous math grade, that correlate with performance expectancies and post-test performance, can be ruled out as alternative explanations. The results are also similar when computing the model with initial performance expectancies as mean of PS1-PE1 and PS1-PE2, summed with PS2-PE1.

6 The results are similar when using merely the sum of PE1-PS1 and PE1-PS2 as initial performance expectancies.
