# Supplementary material for: Performance Expectancies Moderate the Effectiveness of More or Less Generative Activities Over Time
Source: Front Psychol. 2019 Aug 21;10:1623. doi: 10.3389/fpsyg.2019.01623 (PMC6713021; doi:10.3389/fpsyg.2019.01623)
Supplement: Supplementary file 1 [file Data_Sheet_1.docx]

# Appendix A – Materials

Figure A1

*Previous Knowledge Assessment* *- original material presented in German, translation just for demonstration purposes.*


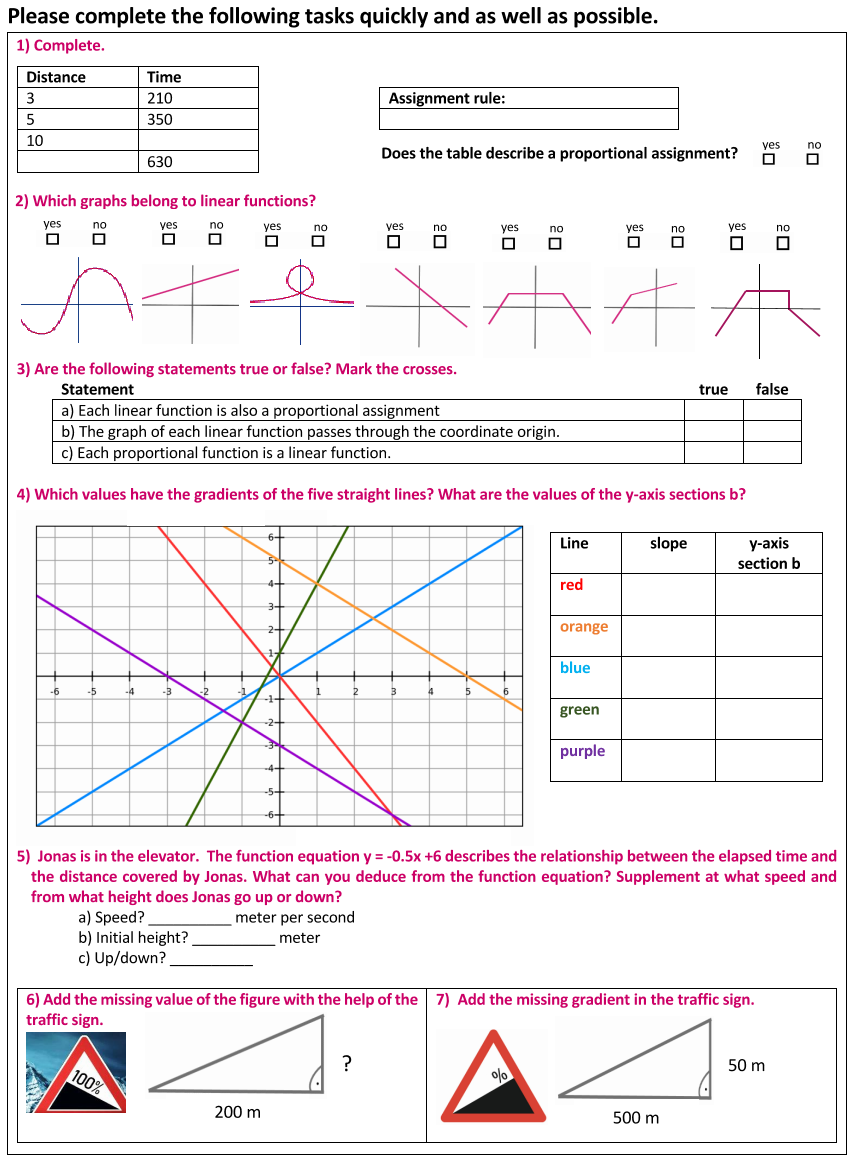


Figure A2

*Summary and Explanatory Material “From Mappings to Functions” - original material presented in German, translation just for demonstration purposes.*


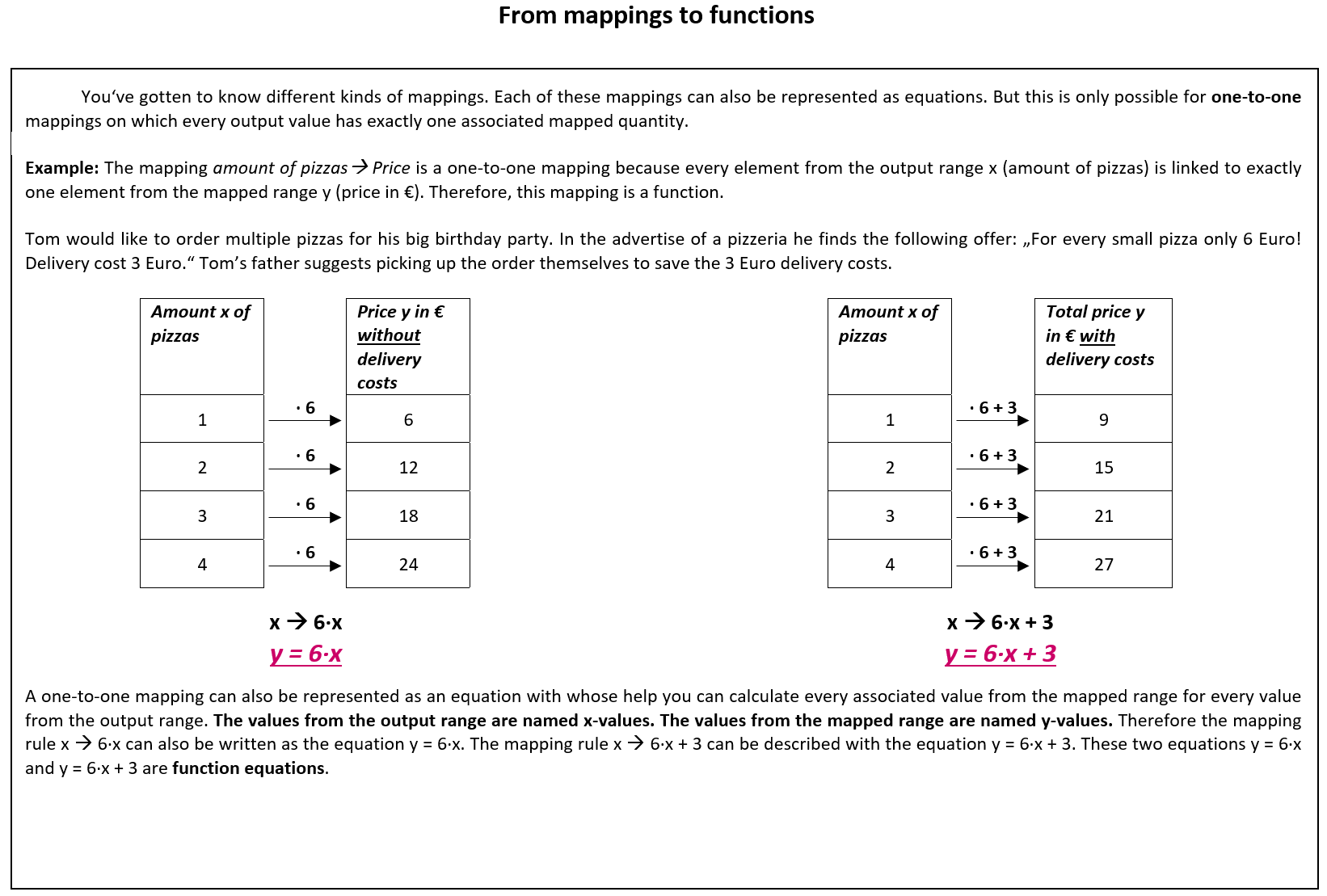

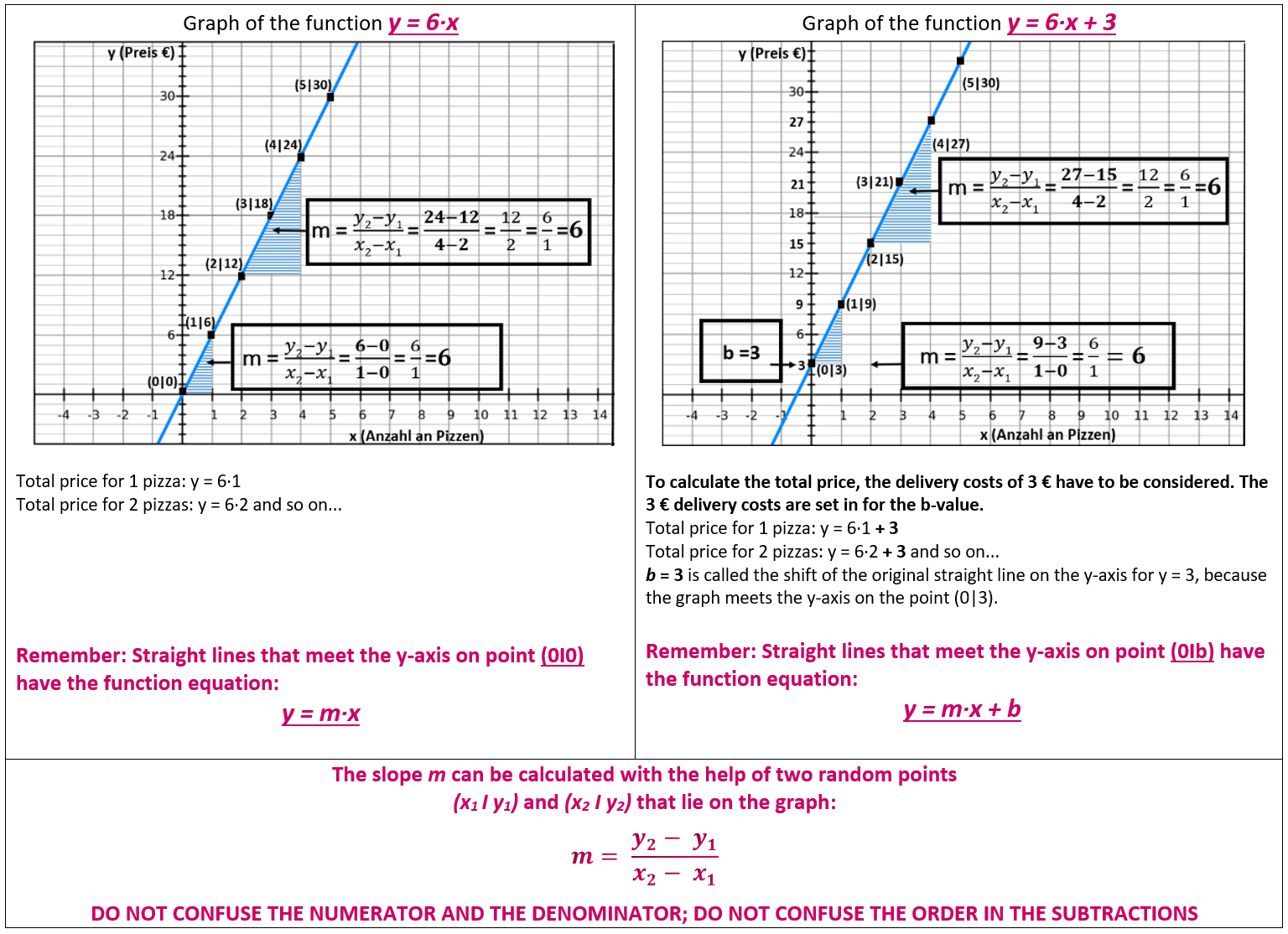


Figure A3

***
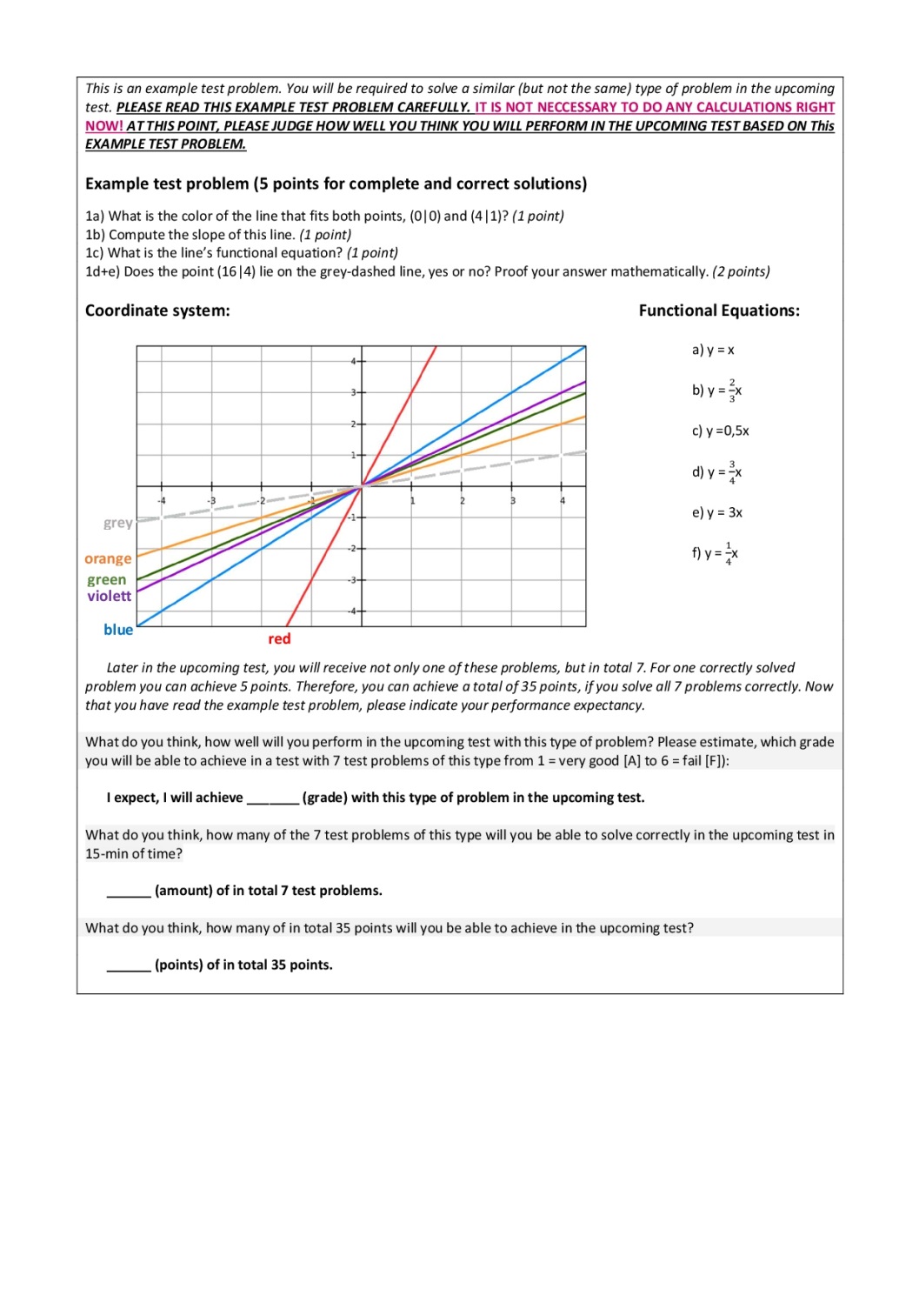
****Measurement of Performance Expectancies Problem Type 1- original material presented in German, translation just for demonstration purposes.*

Figure A4

*Measurement of Performance Expectancies Problem Type 2- original material presented in German, translation just for demonstration purposes.*

*
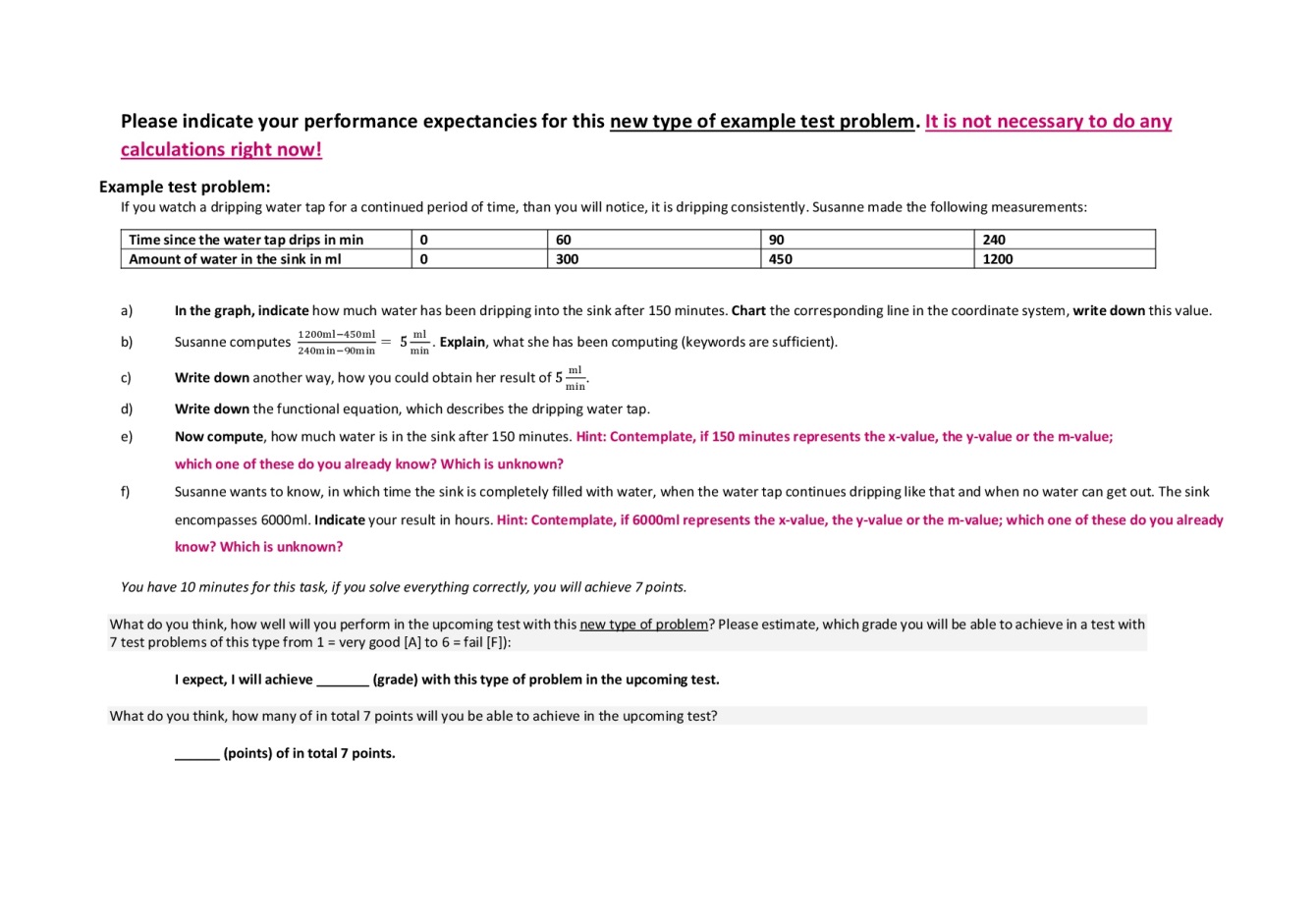
*

Figure A5

*Test Problem Type 1- original material presented in German, translation just for demonstration purposes.*


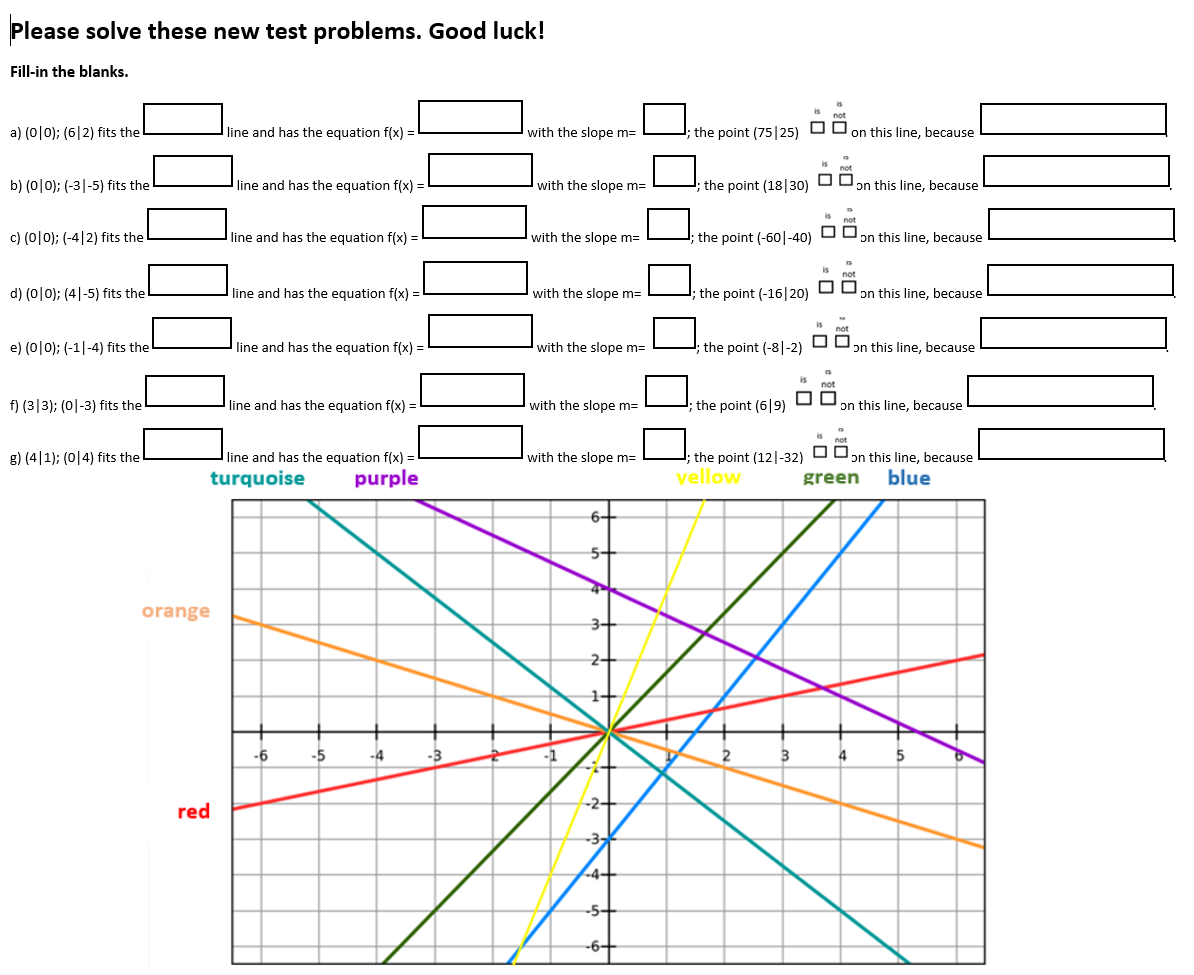


Figure A6

*Test Problem Type 2- original material presented in German, translation just for demonstration purposes.*


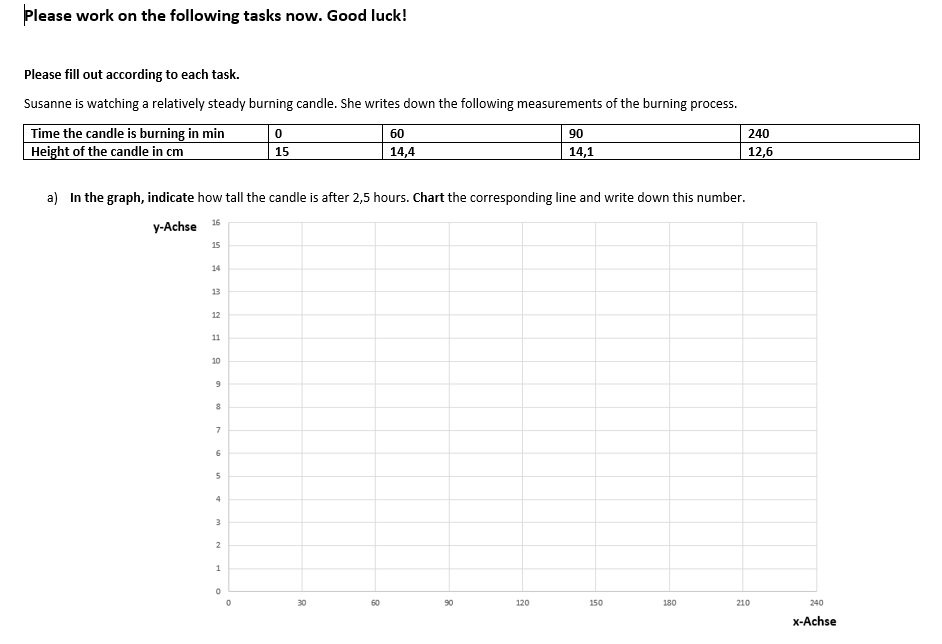


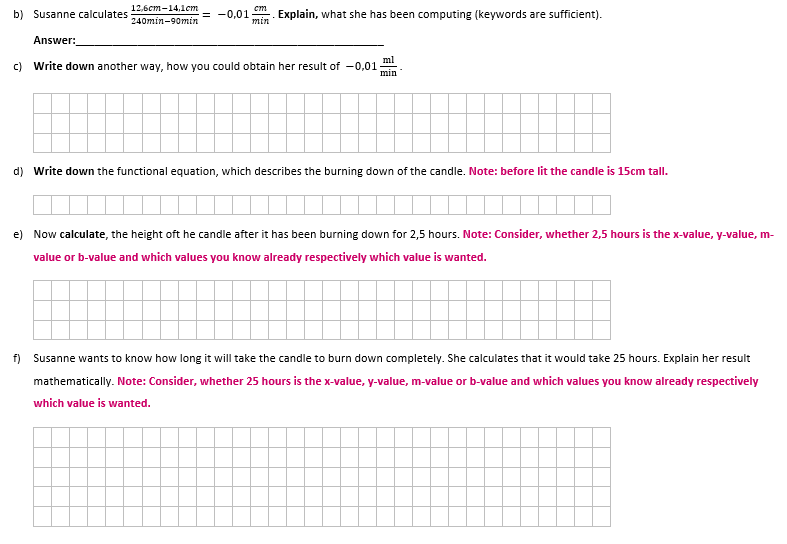


**Appendix B – Control Variables and Additional Analyses**

**Control variables**

As mentioned in the introduction, we also assessed different control variables. Due to readability, detailed descriptions and the results of the analyses adding the control variables follow below (and not in the main text).

We adapted the items from the effort and persistence scale from PISA (2000; Kunter et al., 2002) and collapsed them into one scale (Cronbach’s Alpha = 0.89). The items read, *While working on the learning task…:* “*I worked as diligently as possible*” / “*I tried my best to acquire the knowledge and skills*” / “*I exerted great effort to comprehend all content*” / “*I did not give up even when I experienced difficulties working on the tasks*” / “*I tried my best to do everything as well as possible*” / “*How hard did you try to process the learning task?*”. The items were rated on a 5-point scale from 1 = very little to 5 = very much.

Measuring on a 5-point scale (1 = very little, 5 = very much), we also included the following: “*How difficult was working on the learning task for you?*” / “*Was working on the learning task mentally effortful for you?*”.

After the immediate test, we further asked pupils on 5-point scales (1 = very little to 5 = very much) whether they had had enough time to work on the test problems and how difficult the test problems were.

We also adapted three items from the effort and persistence scale to capture their invested effort in the immediate test (Cronbach’s Alpha = 0.83) and the delayed test (Cronbach’s Alpha = 0.68): assessed on 5-point scale from 1 = very little to 5 = very much collapsed in a scale, “*I tried to solve all test problems as good as possible*” / “*I did not give up even when I experienced the test problems to be very difficult*” / “*I put in much effort to solve the test problems*”.

For exploratory purposes, we assessed participants’ attitude towards and the acceptance of the learning method (solving vs. worked-example). We used the technology acceptance model (TAM; Davis, 1989), which draws on the theory of reasoned action (Ajzen & Fishbein, 1988). The TAM has been used to predict whether students will accept and use, for example, pervasive learning in e-learning (Koondhar et al., 2015). Within the context of the present study, we have adapted the model to test for differences across learning conditions for the model’s components, which allows a prediction about whether students would intend to use solving/worked-examples at home in their self-regulated studies. The items and components of the TAM were adapted to reflect the study’s learning context.

We created nine items to assess perceived usefulness including, “*Learning with the worked-examples/solving exercises helped me to…correctly solve the upcoming test problems/comprehend the upcoming test problems/solve new and unknown test problems/know, which solution step I have to use (even though I may have made a computational mistake)/learn all necessary solution steps/comprehend the learning contents better*”, “*I find this way of learning useful*”, “*I learned how each solution step relates to the other necessary solution steps*”, “*The exercises helped me to exactly understand which solution step has to come next*”, measured on 5-point scales (1=disagreed, 5=agreed); Cronbach’s α = 0.91.

We also created five items to capture perceived ease of use. The items read, “*It was easy for me to work through the worked-examples/solving exercises*.”; “*It was easy to get, what I have to do to calculate the correct problem solution*”; “*Studying with the exercises was easy to do*”; “*Learning with the worked-examples/exercises was too exhausting*”; “*I quickly got used to learning with worked-examples/solving problems*”. All items were assessed by 5-point scales (1=disagreed, 5=agreed). Cronbach’s α = 0.85.

Attitudes were measured by four items, whereby two items pertained to the affective facet and two items covered the cognitive facet of attitudes (see also attitude conceptions by Eagly & Chaiken, 1993; Kruglanski & Stroebe, 2005; Weissgerber, Reinhard, & Schindler, 2017). The affective attitude items were: *“I find it to be pleasant to learn with the previous exercises”*; “*I like learning with the previous exercises”*; the cognitive attitude items were: *“I think that learning with the previous exercises is a good way to comprehend mathematical contents.”*; and, “*I think the previous exercises were a good learning opportunity.”* All ranged from 1=disagree to 5=agree. For this attitude scale, the Cronbach’s α was 0.84.

Four items measured on 5-point scales (1=disagreed, 5=agreed) captured the behavioral usage intention: “*If I am going to prepare for a math test in the future, I would want to learn with these worked-example/solving problems*.”; “*I would welcome it, if we could work more in class with these worked-example/solving problems*.”; “*I always would like to use this way of learning to solve math problems*”; “*Instead of the usual homework assignments, I want more of these worked-example/solving problems in the future.*”; Cronbach’s α = 0.88.

Table 2

*Evaluation of Control Variables across both Learning Conditions.*

| Variable | Condition | | | | |  |  |
| --- | --- | --- | --- | --- | --- | --- | --- |
|  | Worked-examples |  |  | Problem-solving |  |  |  |
|  | *M (SD)* | 95% CI |  | *M (SD)* | 95% CI |  | p-value |
| *Evaluation of both learning conditions after the learning phase^a^* | | | | | | | |
| Exerted effort and persistence scale | 3.92 (0.73) | [3.63; 4.21] |  | 3.70 (1.01) | [3.41; 4.02] |  | 0.34 |
| Task difficulty | 3.16 (1.02) | [2.79; 3.52] |  | 3.55 (1.06) | [3.17; 3.94] |  | 0.14 |
| Mental effort | 3.16 (1.05) | [2.76; 3.55] |  | 3.14 (1.19) | [2.72; 3.55] |  | 0.94 |
| *Evaluation after the immediate Test 1^b^* | | | | | | | |
| Exerted effort and persistence items | 3.56 (1.01) | [3.18; 3.94] |  | 3.62 (1.15) | [3.19; 4.04] |  | 0.84 |
| Test problem difficulty | 3.69 (0.78) | [3.38; 3.99] |  | 3.77 (0.95) | [3.43; 4.11] |  | 0.72 |
| *Evaluation after the delayed test^c^* | | | | | | | |
| Exerted effort and persistence items | 3.56 (0.59) | [3.25; 3.87] |  | 3.47 (1.13) | [3.14; 3.81] |  | 0.72 |
| Test problem difficulty | 3.56 (0.98) | [3.22; 3.90] |  | 3.75 (0.93) | [3.34; 4.11] |  | 0.45 |

*Note.* No significant differences across conditions were obtained.

^a^Using a MANOVA with condition as independent variable and the effort and persistence scale as well as the task difficulty and mental effort item as depend variables yielded no differences across experimental groups *F*(4,57) = 1.47, *p* = 0.21; this also holds for mean comparisons for each variable across conditions (see Table 2).

^b^Using a MANOVA with condition as independent variable and the effort and persistence scale as well as the task difficulty associated with the immediate test as depend variables yielded no differences across experimental groups *F*(2,55) = 0.21, *p* = 0.89; this also holds for mean comparisons for each variable across conditions (see Table 2).

^c^Using the effort and persistence measure and task difficulty associated with the delayed test as dependent variable and condition as independent variable in an MANOVA showed no differences across experimental groups in reported delayed test difficulty or invested effort to solve the test questions, *F*(2, 57) = 0.30, *p* = 0.74, η^2^_p_ = 0.01 (see Table 2).

**Exploratory analyzes for attitudes and acceptance**

Table 3

*Attitudes, Acceptance, and Usage-intention.*

| Variable | Condition | | | | |  |  |
| --- | --- | --- | --- | --- | --- | --- | --- |
|  | Worked-examples |  |  | Problem-solving |  |  |  |
|  | *M (SD)* | 95% CI |  | *M (SD)* | 95% CI |  | p-value |
| Perceived usefulness | 3.44 (0.85) | [3.17; 3.70] |  | 3.26 (0.94) | [2.99; 3.54] |  | 0.39 |
| Perceived ease | 3.24 (0.94) | [2.95; 3.53] |  | 3.19 (1.02) | [2.89; 3.49] |  | 0.82 |
| Attitudes | 3.22 (1.03) | [2.92; 3.51] |  | 3.27 (0.94) | [2.96; 3.57] |  | 0.83 |
| Behavioral intent | 3.25 (1.16) | [2.92; 3.59] |  | 2.96 (1.07) | [2.62; 3.30] |  | 0.23 |

*Note.* No significant differences across conditions were obtained.

We computed a MANOVA with perceived usefulness, perceived ease, attitudes, and usage intention (see Table 2) as dependent variables and condition as independent variables. We found no condition effect, *F*(4,52) = 2.19, *p* = .08, η^2^_p_ = 0.14, only that descriptively worked-examples were evaluated more favorably overall. The mean comparisons for each of the four variables across conditions (see Table 2) showed no significant difference, all *p*s > 0.23.
